# LncRNA VCAN‐AS1 Sponges miR‐374c‐3p to Promote Proliferation, Invasion, Migration, and EMT in Thyroid Cancer

**DOI:** 10.1155/ije/8809262

**Published:** 2026-03-06

**Authors:** Yan Zhang, Lu Sun, Yuqi Wu, Yingzhao Liu, Hui Jiang, Xiaoluo Chen, Li Wang, Chenguang Wu

**Affiliations:** ^1^ Department of Endocrinology, Affiliated People’s Hospital of Jiangsu University, Zhenjiang, Jiangsu, 212000, China, ujs.edu.cn; ^2^ Department of Endocrinology and Metabolism, First Affiliated Hospital of Nanjing Medical University, Nanjing, Jiangsu, 210029, China, njmu.edu.cn; ^3^ The First Clinical Medical College, Nanjing Medical University, Nanjing, Jiangsu, 210029, China, njmu.edu.cn

**Keywords:** EMT, lncRNA VCAN-AS1, miR-374c-3p, thyroid cancer

## Abstract

**Background:**

Thyroid cancer (TC) is the most common endocrine malignancy worldwide, with a rising incidence in recent years. This study investigates the role of long noncoding RNA VCAN‐AS1 (lncRNA VCAN‐AS1) in TC cells and its underlying mechanism.

**Methods and Results:**

LncRNA sequencing and RT‐qPCR revealed that VCAN‐AS1 expression in clinical TC tissues was significantly higher than in paracancerous tissues. Methods such as CCK‐8, cell scratching, Transwell, and Western Blot were employed to assess the impact of VCAN‐AS1 on TC cells. Overexpression of VCAN‐AS1 promoted TC cell proliferation, migration, and invasion; downregulated the epithelial‐mesenchymal transition (EMT)‐related protein E‐cadherin; and upregulated N‐cadherin, vimentin, slug, and snail. Conversely, silencing VCAN‐AS1 reversed these effects. Sequencing identified differentially expressed miR‐374c‐3p in TC cells between the control and VCAN‐AS1 overexpression groups, with functional analysis performed using GO and KEGG pathway enrichment. The regulation of miR‐374c‐3p by VCAN‐AS1 was detected by RT‐qPCR in TC cells, and dual‐luciferase assays confirmed the binding interaction between VCAN‐AS1 and miR‐374c‐3p. VCAN‐AS1 competitively binds with miR‐374c‐3p, and miR‐374c‐3p overexpression counteracts VCAN‐AS1‐induced oncogenic effects. In vivo experiments in mice confirmed the results obtained from in vitro experiments.

**Conclusion:**

VCAN‐AS1 acts as a competing endogenous RNA, driving TC cell proliferation, migration, invasion, and EMT by sponging miR‐374c‐3p. These findings contribute to a novel theoretical basis for TC treatment.

## 1. Introduction

Thyroid cancer (TC) is a predominant endocrine malignancy, exhibiting a higher average incidence in women than in men worldwide and an increasing incidence globally, with a rise in incidence as age increases [1]. With the advancement in medical diagnostics, the incidence of TC has escalated, ranking it ninth among the global cancer incidence rate in 2018 [2]. Although papillary thyroid carcinoma (PTC) is the most prevalent subtype and has a favorable prognosis, some patients still experience metastases to other sites through lymph nodes or other means to other routes, resulting in recurrence or death. Therefore, there is an imperative need to investigate the underlying molecular mechanisms of TC development and identify new therapeutic targets.

Within the human genome, only about 2% of genes encode proteins, while the remaining nontranslated gene portions are referred to as noncoding RNA (ncRNA). Among these, sequences longer than 200 nucleotides are classified as long noncoding RNA (lncRNA) [3]. LncRNAs have been identified as crucial players in various biological processes and are recognized as critical regulators in numerous cancers. They function as either tumor suppressors or oncogenes, exerting significant influence on cancer development and progression [4]. In the context of TC, various lncRNAs play distinct regulatory roles, contributing to the complexity of the regulatory network in TC. For instance, LncRNA MIAT inhibits EZH2 expression through the miR‐150/EZH2 pathway, promoting TC cell invasion [5]. LncRNA MFI2‐AS1, by suppressing miR‐1a‐125p, upregulates TRIAP5 expression, fostering TC development [6]. VCAN antisense ribonucleic acid 1 (VCAN‐AS1) (Gene ID: 105,379,054), an oligosaccharide nucleic acid, is a lncRNA transcribed from a gene located at 5q14.3. Recent studies showed that VCAN‐AS1 promotes the development of gastric cancer [7] and breast cancer [8] and acts mainly through the lncRNA‐miRNA‐mRNA pathway [9]. However, the role of VCAN‐AS1 in TC is unclear. In addition, lncRNAs impact cancer by competitively binding to microRNAs (miRNAs), which are small ncRNAs that inhibit the expression of target genes by degrading mRNA, thereby acting as competing endogenous RNAs (ceRNAs). For instance, LncRNA CALML3‐AS1 functions as a ceRNA for miR‐20a‐5p, thereby regulating the expression of RBM38 and inhibiting the progression of PTC [10].

The process of epithelial‐mesenchymal transition (EMT) is a well‐known contributor to tumor invasion and metastasis, and is regarded as a crucial event in cancer metastasis and a significant indicator of carcinogenesis [11]. EMT is characterized by the loss of E‐cadherin expression, while the elevated expression of mesenchymal markers like N‐cadherin and vimentin is also suggestive of an increased risk of cancer metastasis. In addition, EMT‐associated transcription factors such as zinc finger protein snail and slug exhibited a repressive effect on E‐cadherin transcription, further promoting cancer metastasis [12]. Multiple ceRNA networks targeting the EMT process in TC have been identified. The study indicated that LncRNA TUG1 regulates PTC cell proliferation, migration/invasion, and EMT phenotype establishment by sponging miR‐145 and activating ZEB1 [13]. LINC00460 accelerates PTC progression by modulating the miR‐485–5p/Raf1 axis. Knockdown of LINC00460 inhibits the EMT process by increasing E‐cadherin protein levels and reducing N‐cadherin and vimentin protein levels [14]. In addition, the downregulation of LncRNA CATIP‐AS1 promotes the progression and metastasis of TC through the EMT pathway [15].

To explore the potential molecular mechanisms associated with ceRNA in TC development, identify new therapeutic targets, and examine the role of EMT in this process, this study aimed to explore the role of lncRNA VCAN‐AS1 in TC cell proliferation, invasion, migration, and EMT‐related protein expression. By performing miRNA sequencing to identify differentially expressed miRNAs, we sought to refine and clarify the molecular pathways involved. The results obtained from in vitro experiments were then validated in vivo using a mouse model, thereby confirming the relevance and potential clinical implications of our findings.

## 2. Materials and Methods

### 2.1. Cell Culture

Four cell types were used in this study: Nthy‐ori 3‐1, TPC‐1, BCPAP, and K1 cells. All these were provided by the iCell Bioscience Inc cell bank. Among them, Nthy‐ori 3‐1 were normal human thyroid cells, and TPC‐1, BCPAP, and K1 cells were all human PTC cells. Specifically, TPC‐1 cells harbor a RET/PTC rearrangement, whereas BCPAP cells are characterized by the BRAF^V600E^ mutation. K1 cells are known for their relatively aggressive and invasive phenotype. Nthy‐ori 3‐1, TPC‐1, and BCPAP were cultured in RPMI 1640 medium (Gibco, Shanghai, China) containing 10% fetal bovine serum (FBS), and K1 cells were cultivated in DMEM medium (Gibco, Shanghai, China) containing 10% FBS. All cells were cultured at 37°C in a humidified incubator with 5% CO_2_.

### 2.2. Cell Transfection

PcDNA‐VCAN‐AS1 overexpression vector (VCAN‐AS1) and its negative control (Vector), shRNA against VCAN‐AS1 (VCAN‐AS1 sh1, VCAN‐AS1 sh2) and its negative control (sh‐NC), as well as mimics of miR‐374c‐3p and miR‐4521 and their negative control (miR‐NC) were provided by RiBoBio (Shanghai, China). Cells were seeded in six‐well plates (2 × 10^5^ cells each) and transfected with the corresponding plasmids when cells reached 60% confluence.

### 2.3. Obtaining Clinical Samples

The specimens for this experiment consisted of five pairs of samples, including PTC specimens and adjacent healthy thyroid tissues, surgically excised from five patients at the Department of Thyroid Surgery at the Affiliated People’s Hospital of Jiangsu University. The patients were diagnosed with PTC between September 2019 and October 2021, and subsequently underwent surgery. Exclusion criteria encompassed preoperative radiotherapy or chemotherapy, a history of previous cancer, and the absence of a documented informed consent form. The basic demographic and clinicopathological characteristics of the patients are summarized in Table S1. The control group comprised adjacent thyroid tissues located more than 2 cm away from the edge of PTC. The collection of human tissue specimens and experiments have been approved by the Ethics Committee of the Affiliated People’s Hospital of Jiangsu University (K‐20190062‐Y). All patients included in this trial had preoperative discussions and signed informed consent forms.

### 2.4. LncRNA Sequencing and Analysis

Paired tumor and adjacent nontumorous tissues were collected from patients, and total RNA was extracted using the TRIzol reagent. RNA quality and integrity were assessed by NanoDrop spectrophotometer and Agilent 2100 Bioanalyzer, and samples with RIN ≥ 7.0 were used for sequencing. After rRNA depletion, strand‐specific lncRNA libraries were constructed and sequenced on the DNBSEQ platform (BGI Tech, Shenzhen, China) in paired‐end mode. Clean reads were obtained after quality control and aligned to the human reference genome (GRCh38). Transcript assembly and quantification were performed, and lncRNAs were identified based on transcript length, exon number, and coding potential. Differentially expressed lncRNAs between tumor and normal tissues were screened using DESeq2 with |log_2_Fold Change| ≥ 1 and Q value ≤ 0.05, and the results were visualized using volcano plots. Gene ontology (GO) functional enrichment analysis and Kyoto Encyclopedia of Genes and Genomes (KEGG) pathway enrichment analyses were performed for the differentially expressed lncRNAs using Dr. Tom software, with Q value ≤ 0.05 considered statistically significant.

### 2.5. miRNA Sequencing and Analysis

Total RNA extracted from VCAN‐AS1 overexpressed PTC‐1 cells and control cells was used to construct mRNA sequencing libraries following standard protocols. Briefly, poly (A) + RNA was enriched, fragmented, reverse‐transcribed into cDNA, and ligated with sequencing adapters, followed by PCR amplification. Qualified libraries were sequenced on the DNBSEQ platform. After removing adapter sequences and low‐quality reads, clean reads were mapped to the human reference genome. Gene expression levels were quantified and normalized, and differential mRNA expression between groups was analyzed using DESeq2 with |log2Fold Change| ≥ 1 and Q value ≤ 0.05. Differentially expressed mRNAs were subsequently subjected to GO functional enrichment analysis and KEGG pathway enrichment analysis using Dr. Tom software, and the enriched biological processes and signaling pathways were visualized.

### 2.6. Cell Counting Kit‐8 (CCK‐8) Assay

The CCK‐8 assay was employed to determine the impact of VCAN‐AS1 gene on the activity of TC cells TPC‐1 and BCPAP cells. Around 5 × 10^3^ cells were seeded in 96‐well plates and subjected to incubation for 0, 24, 48, 72, and 96 h. After each time interval, 10 μL of CCK‐8 reagent (BBI Life Sciences) was introduced into each well and kept in a 37°C cell incubator (Thermo) for 1 h in the dark. The absorbance value at 450 nm was detected by a microplate reader (Biotek).

### 2.7. Cell Scratch

To examine the impact of the VCAN‐AS1 gene on the migration of TPC‐1 and BCPAP cells, 2 × 10^5^ cells were inoculated in six‐well plates and incubated for 24 h before transfection. Following transfection, the cells were cultured for an additional 48 h until reaching 90% of confluence. A 200 μL sterile pipette tip was then used to carefully scratch the cell monolayer, and the medium was replaced with DMEM containing 1% FBS for further culturing. The scratch distance was photographed and recorded with a microscope at 0 and 24 h of incubation.

### 2.8. Cell Invasion

After digestion with 0.25% trypsin (Gibco), the cells were suspended and counted. Next, 100 μL of the cells (3 × 10^6^ cells/100 μL) were added to the Trans‐well chamber (CORNING) containing DMEM with 1% FBS, while 600 μL of medium containing 20% FBS was placed in the lower chamber of the 24‐well plate. The Trans‐well chamber was removed after 24 h of culture in the incubator at 37°C, followed by fixation with cell fixative 4% formaldehyde (BBI Life Sciences) for 30 min and stained with 0.1% crystalline violet (BBI Life Sciences) for 30 min. Subsequently, the upper layer of nonmigrating cells was gently wiped off with a cotton swab and washed three times with PBS, and then three randomly selected fields were observed and counted under a 100× microscope (OLYMPUS).

### 2.9. Western Blot

Proteins were extracted from the treated TPC‐1 and BCPAP cells using the RIPA lysis buffer (Beyotime), and the protein concentration was evaluated using the BCA protein assay kit (Beyotime). The proteins were separated by sodium dodecyl sulfate‐polyacrylamide gel electrophoresis (SDS‐PAGE) and transferred onto PVDF membranes. After blocking in TBST containing 5% skim milk for 2 h at room temperature, the membrane was incubated with primary antibodies at 4°C overnight and then cultured with horseradish peroxidase‐conjugated secondary antibodies (anti‐mouse IgG, #7076, or anti‐rabbit IgG, #7074; 1:5000, Cell Signaling Technology, USA) in 37°C for 2 h. The primary antibodies were as follows: anti‐E‐cadherin (1:1000, 20871‐1‐AP, proteintech), anti‐N‐cadherin (1:1000, 13116T, Cell Signaling Technology), anti‐vimentin (1:1000, 60330‐1‐LG, proteintech), anti‐slug (1:1000, ab51772, Abcam), anti‐snail (1:1000, 3879T, Cell Signaling Technology), and anti‐GAPDH (1:10000, 60004‐1‐Ig, proteintech). Then, the ECL Western Blot Kit (CWBIO) was employed for visualization. Protein levels were quantified using the Image *J* software (NIH, Bethesda, MD, USA).

### 2.10. Quantitative Real‐Time PCR (qRT‐PCR)

Total RNA was extracted from five pairs of PTC tissues and adjacent tissues of patients, as well as total RNA from Nthy‐ori 3‐1, TPC‐1, BCPAP, K1 cells, and transfected cells by Trizol (Invitrogen). The first‐strand cDNA was synthesized using the RNA cDNA first‐strand synthesis kit (TransGen) according to the manufacturer’s instructions. Next, reverse transcription was performed using the SG Fast qPCR Master Mix (Sangon Biotech). The amplification conditions were 95°C, 7 s; 57°C, 10 s; and 72°C, 15 s, a total of 45 cycles. Glyceraldehyde 3‐phosphate dehydrogenase (GAPDH) was used as the endogenous control of VCAN‐AS1, while U6 was used as the endogenous control for lncRNA and miRNA. The primer sequences for the detected lncRNA are listed in Table 1, and the primer sequences for the other molecules are listed in Table 2.

**TABLE 1:** Primer sequences for human lncRNAs used in Real‐Time qPCR.

Gene	Primer sequences (forward/reverse)
H‐GAPDH	5’ ‐ GGA​GCG​AGA​TCC​CTC​CAA​AAT ‐ 3′5’ ‐ GGC​TGT​TGT​CAT​ACT​TCT​CAT​GG ‐ 3′
H‐VCAN‐AS1	5’ ‐ GCC​ACA​TCA​CAG​CTG​ACA​TAC‐ 3′ 5’ ‐ GCC​ACC​AAC​ATA​CTT​GAC​AGA ‐ 3′
H‐LINC01614	5’ ‐ TGA​CCC​AAC​ACT​CAG​TCC​AC ‐ 3′ 5’ ‐ CAA​TGC​AGA​CTT​GCT​CCC​AG ‐ 3′
H‐TMEM92‐AS1	5’ ‐ GAA​GTC​TCG​GCG​GGA​GAT​TA ‐ 3′ 5’ ‐ AGG​TCT​GTC​TTG​GGG​TCT​CC ‐ 3′
H‐lncRNA CRNDE	5’ ‐ CGA​TCG​CGC​TAT​TGT​CAT​GG ‐ 3′ 5’ ‐ TCC​GCC​TCG​CTT​AGA​CAT​TG ‐ 3′
H‐LINC00261	5’ ‐ AGG​CCG​TGA​AGC​TAA​AGG​TC ‐ 3′ 5’ ‐ GTG​AGC​CGA​GAT​GAA​CAG​GT ‐ 3′
H‐MPPED2‐AS1	5’ ‐ GGA​CCC​CAT​CAG​CCT​TGA​AA ‐ 3′ 5’ ‐ TCA​CTG​GTG​AAC​GAC​TGC​AA ‐ 3′
H‐F11‐AS1	5’ ‐ GCT​CGT​GAT​GCC​TAC​CAG​AT ‐ 3′ 5’ ‐ GTT​GGA​TGA​GGA​GTT​AGC​GGT ‐ 3′
H‐HNF‐1A AS1	5’ ‐ TCAAGAAATGGTGGCTAT ‐ 3′ 5’ ‐ GCTCTGAGACTGGCTGAA ‐ 3′

**TABLE 2:** Primer sequences for human miRNAs used in Real‐Time qPCR.

Gene	Primer sequences (forward/reverse)
H‐miR‐374c‐3p	5’ ‐ CAC​TTA​GCA​GGT​TGT​ATT​ATA​T ‐ 3′
H‐miR‐4521	5’ ‐ GCT​AAG​GAA​GTC​CTG​TGC​TCA​G ‐ 3′
U6	5’ ‐ CTCGCTTCGGCAGCACA ‐ 3′
	5’ ‐ AAC​GCT​TCA​CGA​ATT​TGC​GT ‐ 3′

### 2.11. Dual‐Luciferase Assay

To investigate the binding relationship between miRNA374c‐3p and VCAN‐AS1, the dual luciferase activity assay was performed. The luciferase reporter vector containing the VCAN‐AS1‐MUT sequence was provided by Promega (Madison, WI, USA). The plasmid was transfected by a lipo2000 transfection kit and detected using the Dual‐Luciferase Reporter Assay kit (Promega) following the instructions of the manufacturer.

### 2.12. Animal Experiments

Fifteen male BALB/c nude mice (6–8 weeks, 18–20 g) were obtained from Sipeifu (Beijing, China). The mice were randomly divided into three groups (five mice per group): NC group, VCAN‐AS1‐OE group, and VCAN‐AS1‐OE + miR‐374c‐3p mimic group. TC cell line BCPAP was transfected with lentivirus‐packaged pcDNA empty vector (NC group) and pcDNA‐VCAN‐AS1 overexpression vector (VCAN‐AS1‐OE group and VCAN‐AS1‐OE + miR‐374c‐3p mimic group). Following cell stable transfection, 5 × 10^6^ cells were subcutaneously injected into the right flank of the mice. When the tumors reached a visible size, 2 × 10^6^ transducing units of lentivirus‐miR‐374c‐3p mimic in 50 μL PBS was injected into the tumors of the VCAN‐AS1‐OE + miR‐374c‐3p mimic group every 3 days, while the other groups received equal volumes of lentivirus‐miR‐NC (Ribobio, Shanghai, China). The mice were housed at 22°C–24°C under specific pathogen‐free conditions with a 12‐h light/dark cycle and had free access to food and water. From day 7 to day 34, body weight and tumor volume were measured every 3 days, using formula V = Length × width^2^/2. After 34 days of treatment, the mice were euthanized under excessive isoflurane anesthesia, and the tumors were excised, weighed, and measured for volume before imaging. Tumor homogenates were analyzed via Western blot for the expression of E‐cadherin, N‐cadherin, vimentin, slug, snail, and GAPDH, and RT‐qPCR was used to assess the level of miR‐374c‐3p in tumors. The experiments were approved by the Ethics Committee of the Affiliated People’s Hospital of Jiangsu University (K‐20190062‐Y) and were conducted in compliance with the ARRIVE guidelines, as well as Guidance on the operation of the Animals (Scientific Procedures) Act 1986 and the NIH Guide for the Care and Use of Laboratory Animals.

### 2.13. Statistical Analysis

All experiments were conducted in triplicate and the results presented as means ± standard deviations. Statistical analyses were performed using GraphPad Prism 7. The normality of the data was evaluated prior to applying univariate ANOVA to compare the results between multiple groups. For pairwise comparisons between two groups, Student’s *t*‐test was utilized. A *p* value of less than 0.05 was considered to indicate statistical significance.

## 3. Results

### 3.1. Analysis and Validation of Differentially Expressed Genes in TC

In this study, we performed lncRNA sequencing on patients’ TC tissues and adjacent normal tissues, and screened for differentially expressed genes. The volcano plot of differentially expressed genes (Figure 1(a)) showed that there were 1888 upregulated lncRNAs and 878 downregulated lncRNAs in TC. KEGG pathway enrichment analysis (Figure S1A) showed that differentially expressed lncRNAs were mainly concentrated in cellular processes, environmental information processing, genetic information processing, human diseases, metabolism, and organismal systems–related pathways. The GO cellular component enrichment bubble plot (Figure S1B) showed that differentially expressed lncRNAs in TC were mainly enriched in membrane, integral component of membrane, plasma membrane, extracellular region, extracellular space, and other cell components. Subsequently, RT‐qPCR experiments were performed to detect the expression of several significantly differentially expressed and of interest lncRNAs (four upregulated and four downregulated) in cancer tissues and paracancerous tissues to validate the sequencing results. Figures 1(b), 1(c), 1(d), 1(e), 1(f), 1(g), 1(h), and 1(i) showed that VCAN‐AS1, LINC01614, TMEM92‐AS1, and lncRNA CRNDE were significantly upregulated in TC compared to normal tissues (*p* < 0.001), LINC00261 and MPPED2‐AS1 were significantly downregulated in TC compared to normal tissues (*p* < 0.05), and F11‐AS1 and HNF‐1A AS1 did not show significant changes. It is noteworthy that the alteration in VCAN‐AS1 is particularly pronounced in both sequencing and RT‐qPCR results, and its role in TC remains unexplored, prompting an in‐depth investigation into the impact of VCAN‐AS1 on TC.

FIGURE 1Identification and validation of differentially expressed lncRNAs in thyroid cancer. (a) Volcano plot of differentially expressed genes. Arrow indicates LncVCAN‐AS1. (B‐I) RT‐qPCR was performed to detect the mRNA expression of VCAN‐AS1 (b), LINC01614 (c), TMEM92‐AS1 (d), lncRNA CRNDE (e), LINC00261 (f), MPPED2‐AS1 (g), F11‐AS1 (h), and HNF‐1A AS1 (i) in thyroid cancer tissues and paracancerous tissues. ^∗^ *p* < 0.05, ^∗∗∗^ *p* < 0.001.

**Figure:**
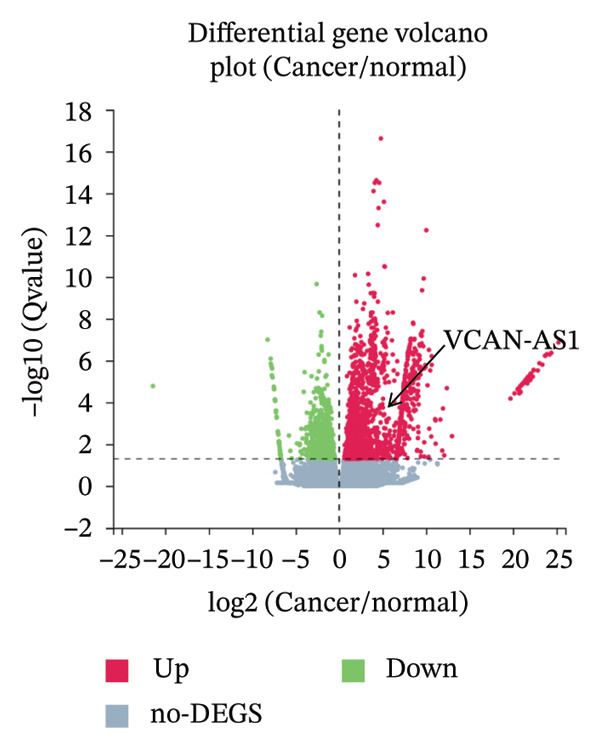
(a)

**Figure:**
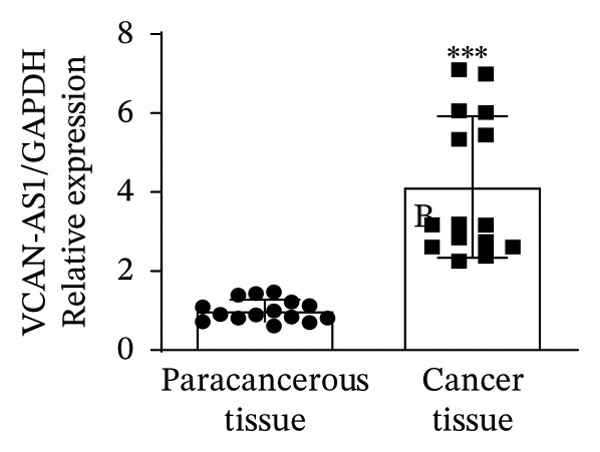
(b)

**Figure:**
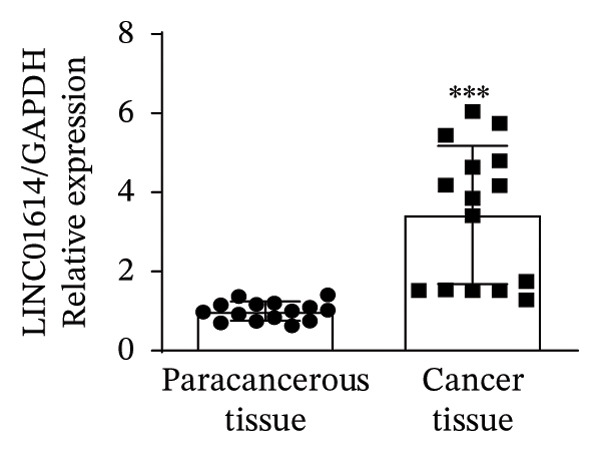
(c)

**Figure:**
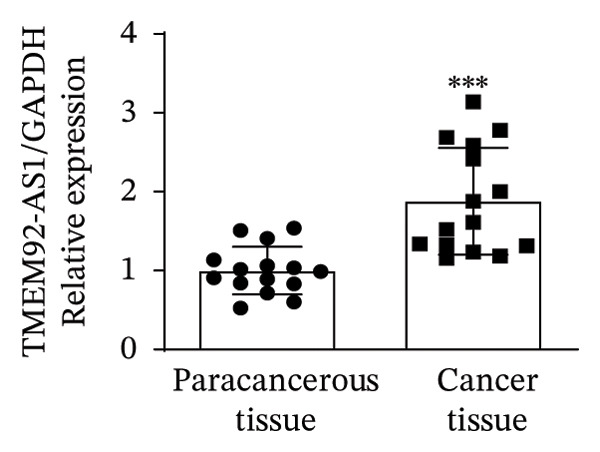
(d)

**Figure:**
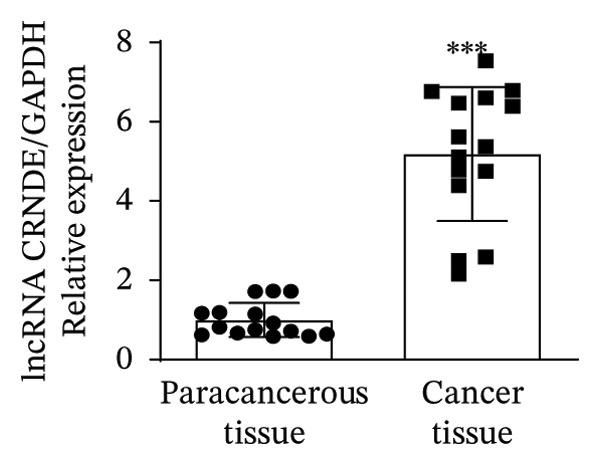
(e)

**Figure:**
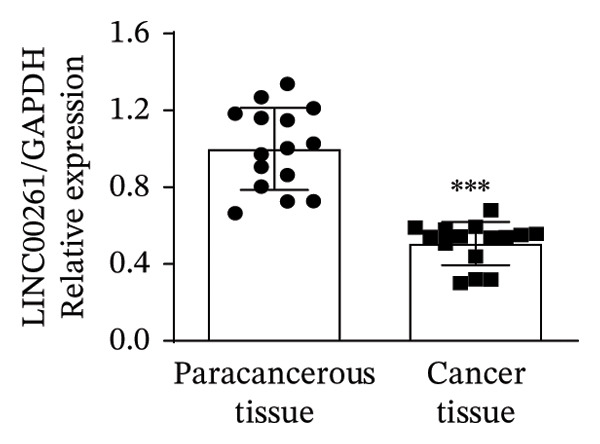
(f)

**Figure:**
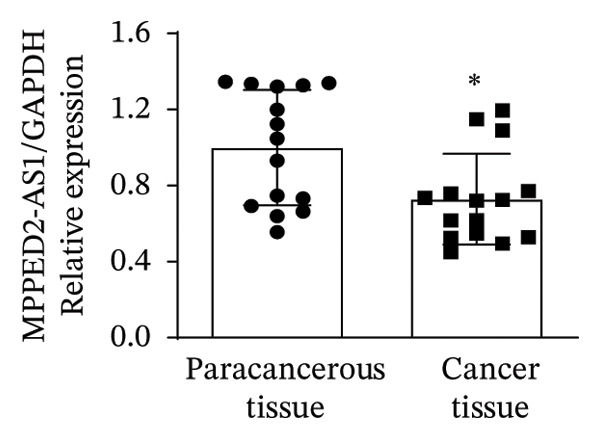
(g)

**Figure:**
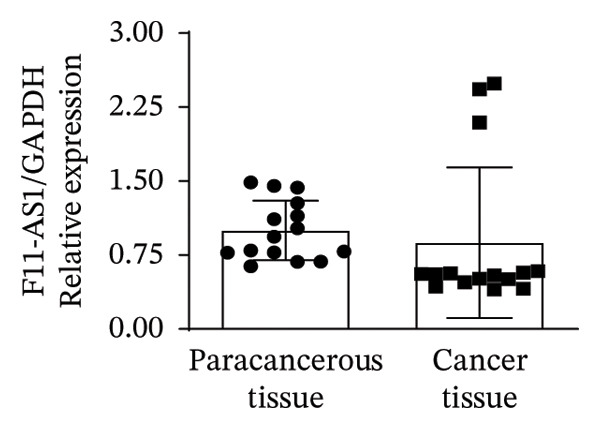
(h)

**Figure:**
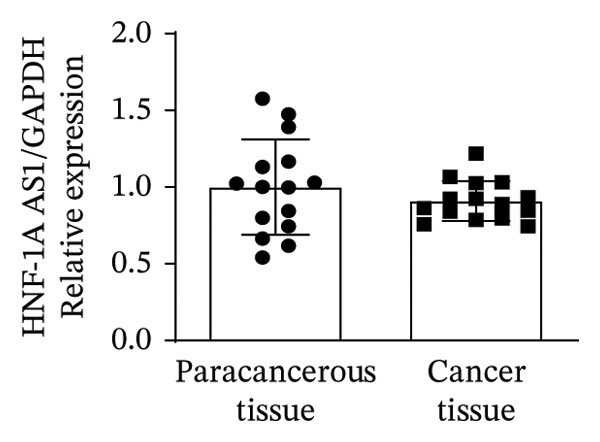
(i)

### 3.2. Effect of VCAN‐ASI Overexpression or Silencing on TC Cells

The RT‐qPCR was carried out to detect the expression of VCAN‐AS1 in the three TC cells, TPC‐1, BCPAP, and K1. The results showed that the mRNA expression of VCAN‐AS1 was elevated in TC cells compared to normal thyroid cells (*p* < 0.01), and the VCAN‐AS1 expression was the highest in BCPAP cells, followed by TPC‐1 cells (Figure 2(a)). The transfection efficiency validation results show that VCAN‐AS1 is effectively overexpressed in normal thyroid cells after transfection with the overexpressed plasmid and successfully downregulated in BCPAP cells after transfection with shRNA (Figure 2(b)). The VCAN‐AS1‐overexpressed TC cells were obtained by transfection with overexpressed vectors. The CCK‐8 assay revealed that the cell viability of both TPC‐1 cells and BCPAP cells in the VCAN‐AS1 OE group was higher than that in the vector group and blank group (*p* < 0.001) (Figures 2(c), 2(d)). Similarly, in the Trans‐well (Figures 2(e), 2(f), 2(i)) and cell scratch assays (Figures 2(g), 2(h), 2(j)), the VCAN‐AS1 OE group had higher cell invasion and migration capacity than the vector and blank groups (*p* < 0.001). In addition, enlarged representative images are provided in the supporting figures to further illustrate EMT‐associated morphological changes induced by VCAN‐AS1 overexpression. Specifically, VCAN‐AS1‐overexpressed BCPAP cells exhibited a pronounced spindle‐shaped and elongated morphology, while TPC‐1 cells appeared elongated and narrowed, displaying a fibroblast‐like phenotype with reduced intercellular adhesion (Figure S2). Furthermore, the results of the Western Blot (Figures 2(k), 2(l)) showed that the E‐cadherin protein expression was significantly reduced in VCAN‐AS1‐overexpressed BCPAP cells and TPC‐1 cells, whereas the expression of N‐cadherin, vimentin, slug, and snail was prominently increased, indicating the proceeding of EMT and a rise in the metastatic capacity of TC cells.

FIGURE 2Effect of overexpressed VCAN‐AS1 on TPC‐1 and BCPAP cells. (a) RT‐PCR detection of VCAN‐AS1 expression in normal thyroid cells Nlthy‐sri 3‐1 and in 3 TC cells (TPC‐1, BCPAP, K1). (b) RT‐PCR verifies VCAN‐AS1 overexpression in normal thyroid cells and downregulation in TC cells. (c), (d) CCK‐8 assay detecting the impact of VCAN‐AS1 OE on the proliferation of TPC‐1 and BCPAP cells. (e), (f) Trans‐well assay determining the impact of VCAN‐AS1 OE on the invasion of TPC‐1 and BCPAP cells. (g), (h) Cell scratch assay detecting the impact of VCAN‐AS1 OE on the migration of TPC‐1 and BCPAP cells. (i) Relative cell invasion in the Transwell assay. (j) Migration rate in the wound healing assay. (k), (l)Western Blot testing of the impact of VCAN‐AS1 OE on the EMT‐related protein expression of TPC‐1 and BCPAP cells. Data in the figure represent mean ± standard deviation, ^∗^ *p* < 0.05, ^∗∗^ *p* < 0.01, ^∗∗∗^ *p* < 0.001, *n* = 3.

**Figure:**
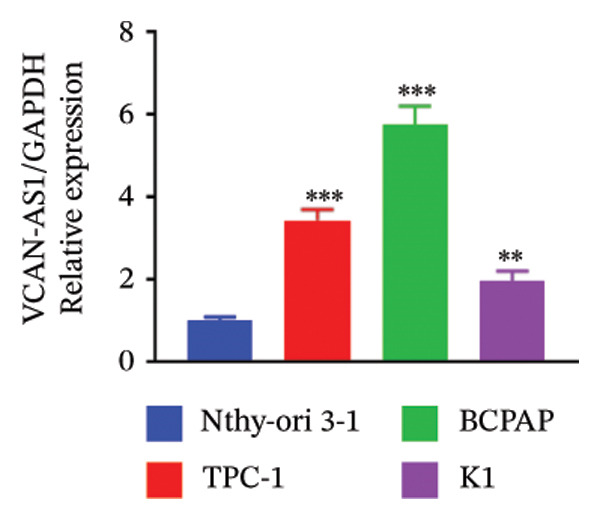
(a)

**Figure:**
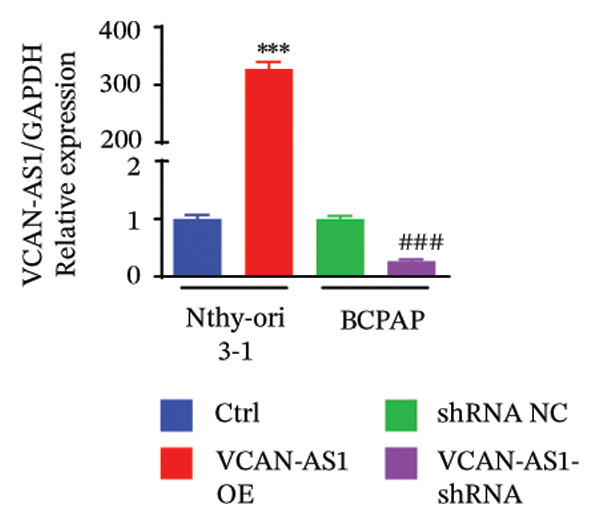
(b)

**Figure:**
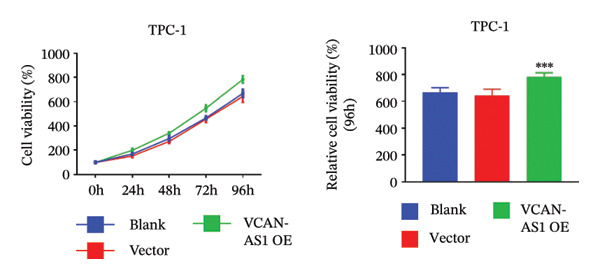
(c)

**Figure:**
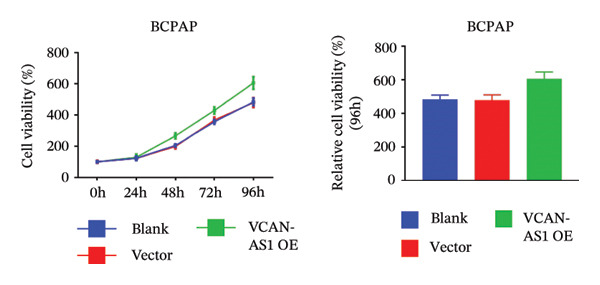
(d)

**Figure:**
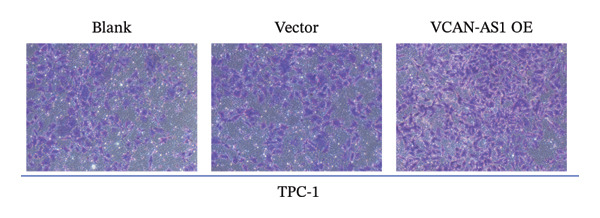
(e)

**Figure:**
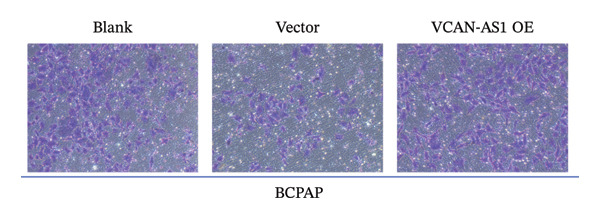
(f)

**Figure:**
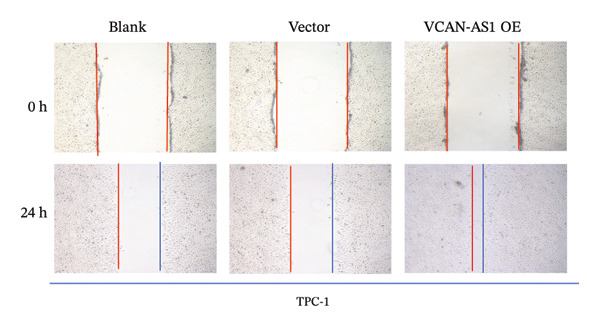
(g)

**Figure:**
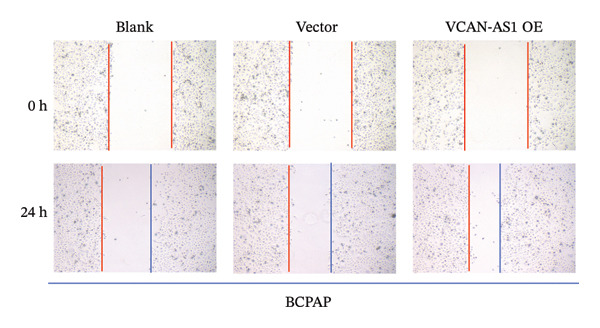
(h)

**Figure:**
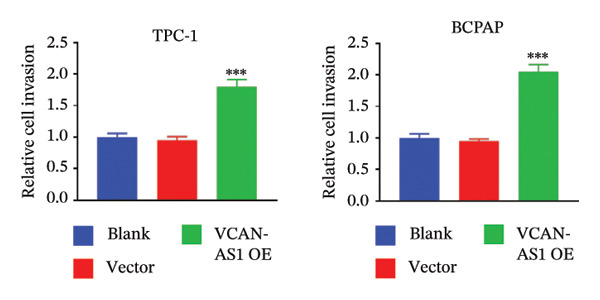
(i)

**Figure:**
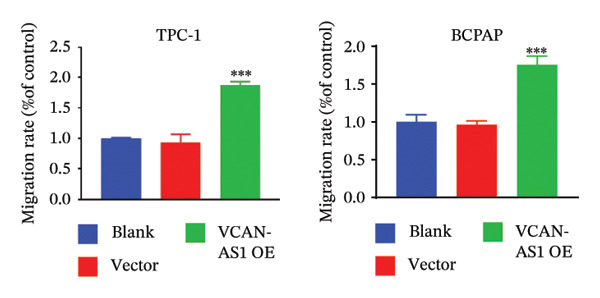
(j)

**Figure:**
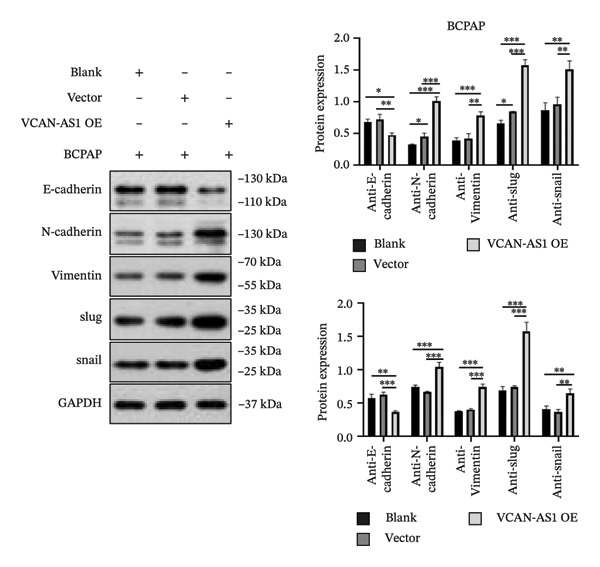
(k)

**Figure:**
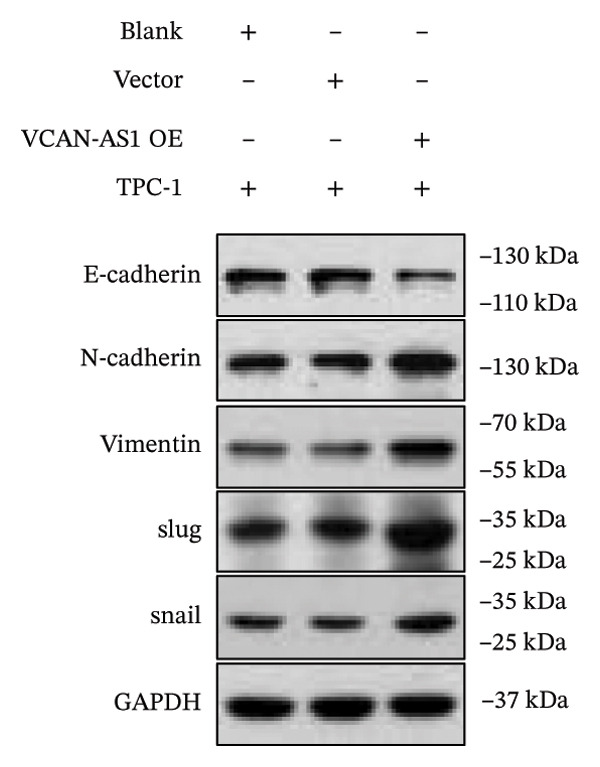
(l)

Silencing of VCAN‐AS1 in TPC‐1 and BCPAP cells was achieved through transfection with VCAN‐AS1 sh1 and VCAN‐AS1 sh2. According to the results of CCK‐8 (Figures 3(a), 3(b)), the cell viability of TPC‐1 cells was reduced after silencing VCAN‐AS1 (*p* < 0.05), and the VCAN‐AS1 sh1 group had a more pronounced decrease in cell activity than the VCAN‐AS1 sh2 group. While in BCPAP cells, VCAN‐AS1 silencing also inhibited cell viability (*p* < 0.01), and the VCAN‐AS1 sh1 group had a similar inhibitory effect to the VCAN‐AS1 sh2 group. Furthermore, Trans‐well (Figures 3(c), 3(d), 3(g)) and cell scratch assays (Figures 3(e), 3(f), 3(h)) showed that silencing VCAN‐AS1 suppressed the invasion and migration of TPC‐1 and BCPAP cells (*p* < 0.01), with the VCAN‐AS1 sh1 group exhibiting a more significant inhibitory effect (*p* < 0.001). Meanwhile, the Western Blot (Figures 3(i), 3(j)) indicated that after silencing VCAN‐AS1, the protein expression of E‐cadherin in BCPAP and TPC‐1 cells was remarkably elevated (*p* < 0.05), while the protein expression of N‐cadherin, vimentin, slug, and snail was dramatically diminished (*p* < 0.01), implying that silencing lncRNA could impede EMT and the VCAN‐AS1 sh2 group had a more significant inhibitory effect.

FIGURE 3Effect of silencing VCAN‐AS1 on TPC‐1 cells and BCPAP cells. (a), (b) CCK‐8 assay examining the impact of VCAN‐AS1 sh1 and VCAN‐AS1 sh2 on the proliferation of the 2 TC cells. (c), (d) Trans‐well assay detecting the impact of VCAN‐AS1 sh1 and VCAN‐AS1 sh2 on the invasion of the 2 TC cells. (e), (f) Cell scratch assay determining the impact of VCAN‐AS1 sh1 and VCAN‐AS1 sh2 on the migration of the 2 TC cells. (g) Relative cell invasion in the Transwell assay. (h) Migration rate in the wound healing assay. (i), (j) Western Blot examining the impact of VCAN‐AS1 sh1 and VCAN‐AS1 sh2 on the EMT‐related protein expression of the 2 TC cells. Data in the figure represent mean ± standard deviation, ^∗^ *p* < 0.05, ^∗∗^ *p* < 0.01, ^∗∗∗^ *p* < 0.001, *n* = 3.

**Figure:**
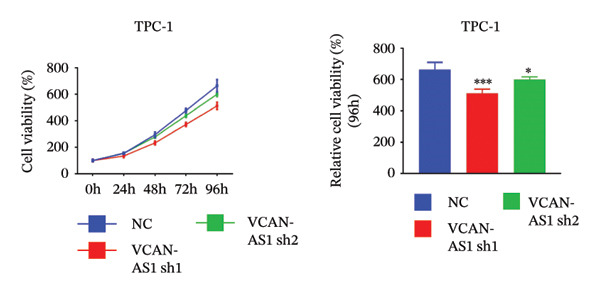
(a)

**Figure:**
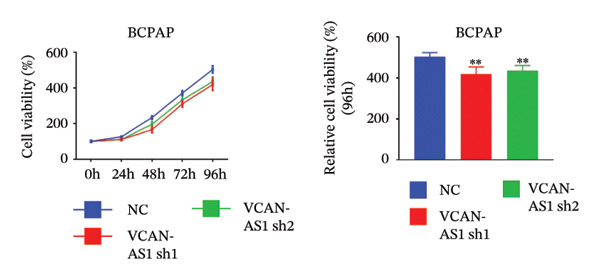
(b)

**Figure:**
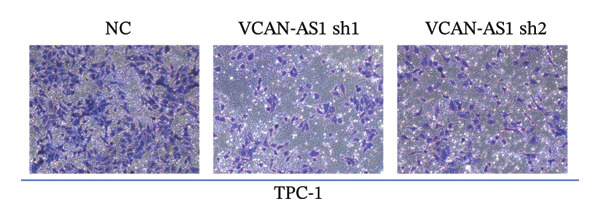
(c)

**Figure:**
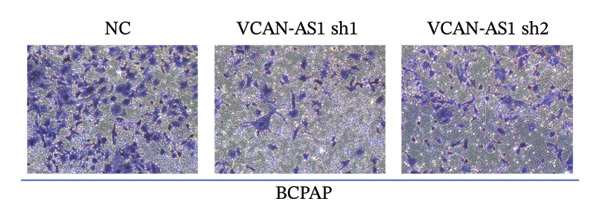
(d)

**Figure:**
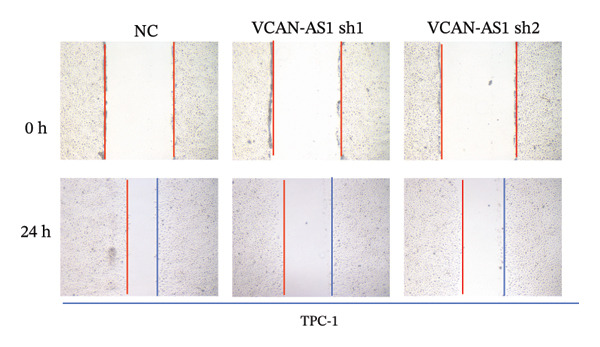
(e)

**Figure:**
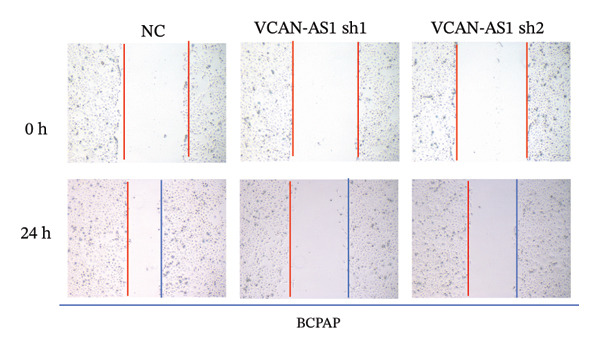
(f)

**Figure:**
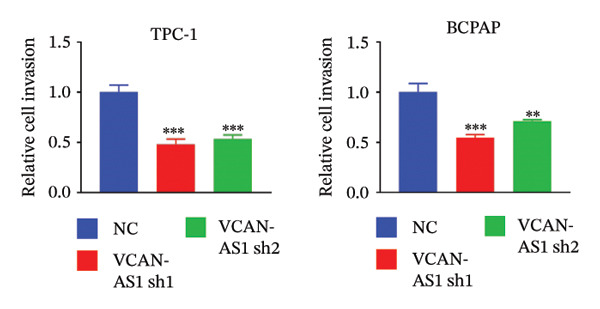
(g)

**Figure:**
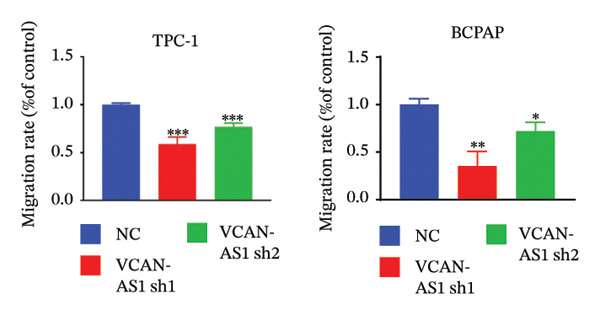
(h)

**Figure:**
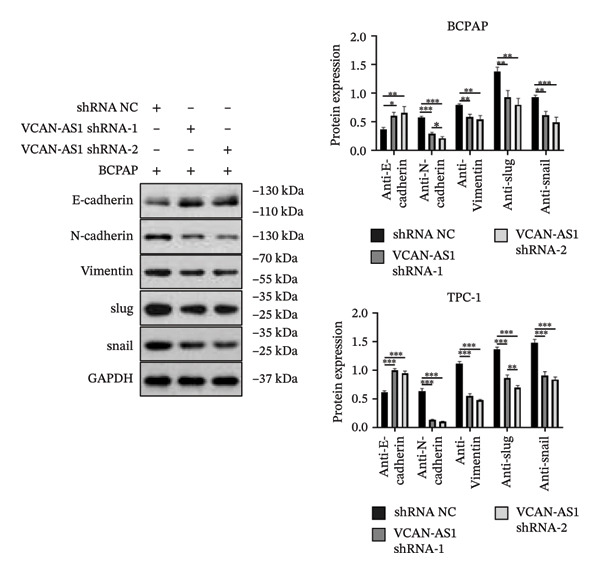
(i)

**Figure:**
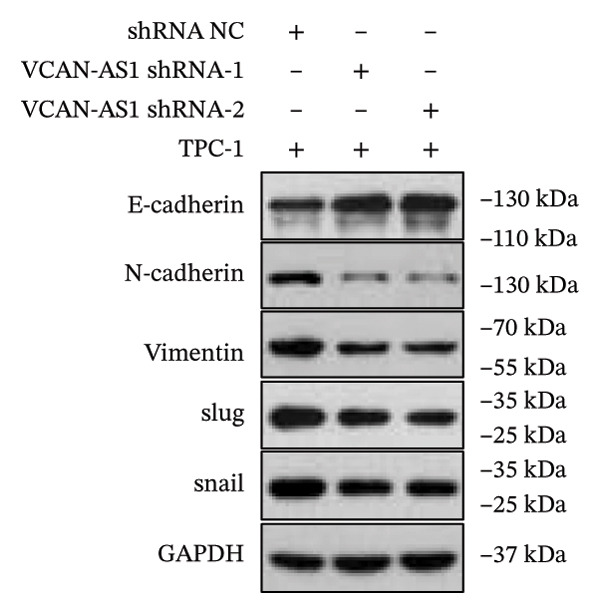
(j)

In summary, lncRNA‐VCAN‐AS1 overexpression stimulated the proliferation, invasion, and migration of TC cells; amplified the expression of N‐cadherin, vimentin, slug, and snail; and impeded the expression of E‐cadherin, thereby fortifying EMT. Conversely, the silencing of lncRNA‐VCAN‐AS1 could repress the viability of TC cells and impede cell proliferation, invasion, migration, and EMT.

### 3.3. KEGG Pathway and GO Term Enrichment Analysis of miR‐374c‐3p

Utilizing a differential gene clustering heatmap (Figure 4(a)), we compared the differential expression of different miRNAs between the negative control cells (ctrl group) and the VCAN‐AS1 OE cells (treat group). The most significant differential expression was observed in miR‐374c‐3p, followed by miR‐4521. In the ctrl group, these miRNAs showed lower abundance, while in the treatment group, their abundance was higher (Table S2). This suggests a potential correlation between VCAN‐AS1 and miR‐374c‐3p. To explore the function of miR‐374c‐3p, both GO and KEGG pathway enrichment analyses were conducted. GO cellular component analysis (Figure 4(b)) showed that the predicted target genes of miR‐374c‐3p were mainly enriched in intracellular components, including nuclear‐ and cytoplasm‐associated regions, as well as cytoskeletal structures. In addition, KEGG pathway enrichment analysis (Figure 4(c)) identified the top 20 significantly enriched pathways associated with the predicted target genes of miR‐374c‐3p. These pathways were primarily involved in signal transduction, cancer‐related pathways, endocrine system‐associated pathways, and immune‐related pathways.

FIGURE 4Differentially expressed gene miR‐374c‐3p and its gene function. (a) MiRNA sequencing of samples from the ctrl group and the treat group. (b) GO enrichment analysis of miR‐374c‐3p. (c) KEGG pathway enrichment analysis of miR‐374c‐3p.

**Figure:**
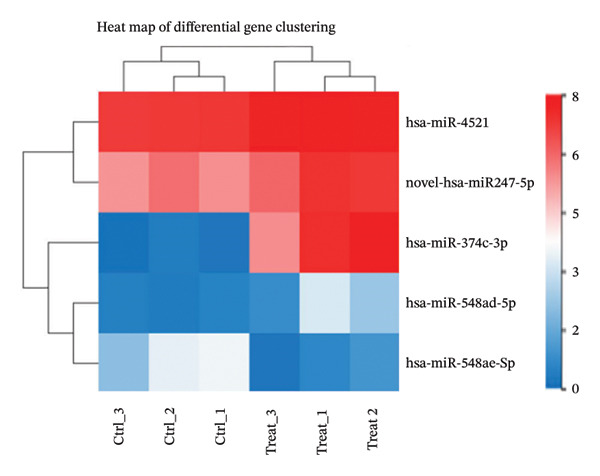
(a)

**Figure:**
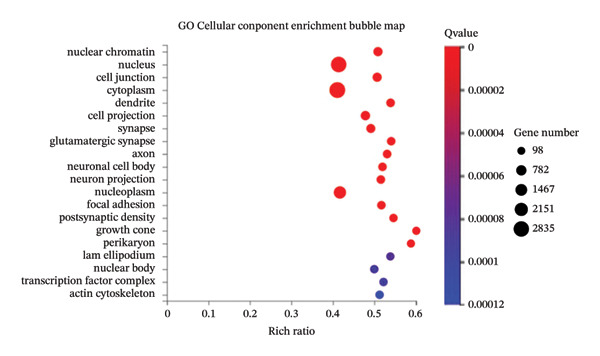
(b)

**Figure:**
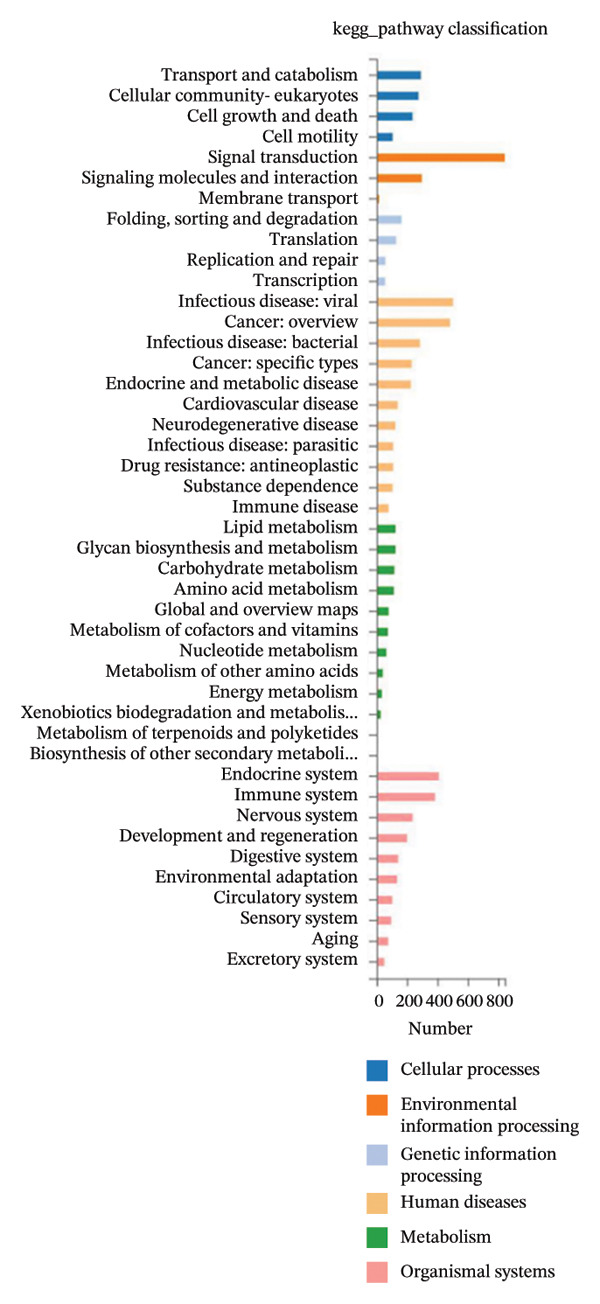
(c)

### 3.4. The Effect of lncRNA‐VCAN‐AS1 Expression on miR‐374c‐3p and miR‐4521

To further investigate the mechanism of action of lncRNA VCAN‐AS1, we selected the BCPAP cells with the highest expression of VCAN‐AS1 to examine the expression profiles of differentially expressed miRNAs. The results of RT‐PCR results indicated that overexpression of VCAN‐AS1 led to a reduction in the expression of miR‐374c‐3p and miR‐4521 in normal thyroid cells (*p* < 0.01). Conversely, silencing VCAN‐AS1 resulted in upregulation of the expression of miR‐374c‐3p and miR‐4521 in BCPAP cells (*p* < 0.05). It is worth noting that the regulation of miR‐374c‐3p by VCAN‐AS1 was more prominent (*p* < 0.001), as depicted in Figures 5(a), 5(b).

FIGURE 5Aberrant expression of VCAN‐AS1 in TC cells and its target miRNA. (a), (b) RT‐PCR examining the impact of VCAN‐AS1 on miR‐374c‐3p and miR‐4521 in normal thyroid cells and TC cells. (c) The predicted seed recognition sites between the 3′UTR of VCAN‐AS1 and miR‐374c‐3p are marked in red. (d) Dual luciferase activity assay validating miR‐374c‐3p and VCAN‐AS1. Data in the figure represent mean ± standard deviation, ^∗∗^ *p* < 0.01, ^∗∗∗^ *p* < 0.001, ^#^ *p* < 0.05, ^###^ *p* < 0.001, *n* = 3.

**Figure:**
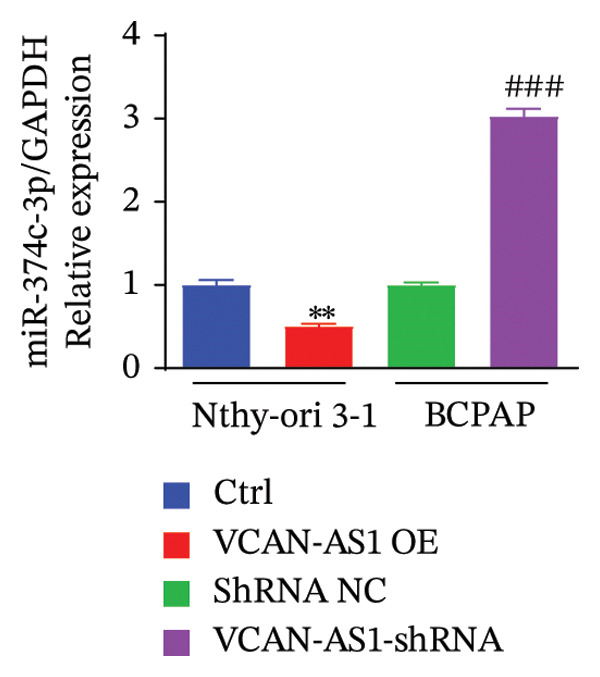
(a)

**Figure:**
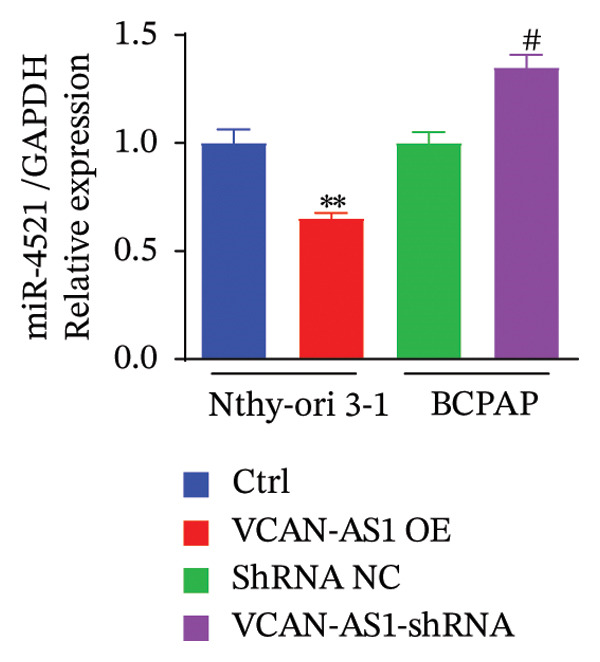
(b)

**Figure:**
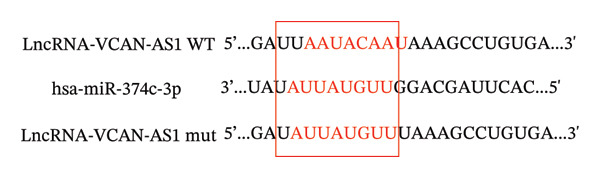
(c)

**Figure:**
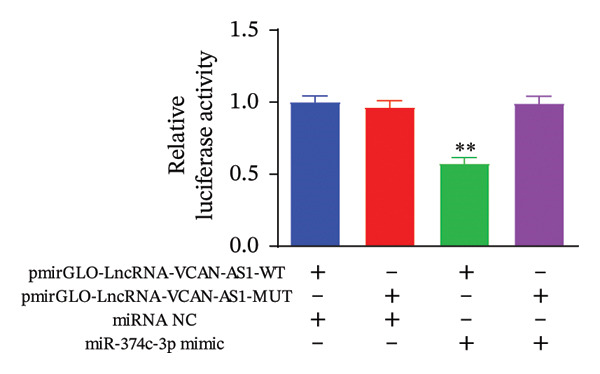
(d)

### 3.5. VCAN‐AS1 Targeting miR‐374c‐3p

As displayed in Figures 5(c), 5(d), a dual luciferase assay on BCPAP cells co‐transfected with the cloning vector and miR‐374c‐3p mimics showed that the luciferase activity of lncRNA‐VCAN‐AS1‐wt was significantly reduced (*p* < 0.01), whereas the luciferase activity of lncRNA‐VCAN‐AS1‐mut did not change markedly, indicating that lncRNA VCAN AS1 could play a role similar to ceRNA through direct sponge adsorption of miR‐374c‐3p.

### 3.6. Effect of VCAN‐AS1 and miR‐374c‐3p on TPC‐1 and BCPAP Cells

The plasmid overexpressing VCAN‐AS1 and the miR‐374c‐3p mimics were transfected into TPC1 and BCPAP cells for experimentation. RT‐PCR analysis, as shown in Figure 6(a), indicated a significant upregulation in the expression of miR‐374c‐3p in the VCAN‐AS1‐OE + miR‐374c‐3p mimic group. The results obtained from the CCK‐8 assay suggested that the increased expression of miR‐374c‐3p significantly inhibited the cell viability of TPC‐1 and BCPAP cells (*p* < 0.01) (Figures 6(b), 6(c)) while hampering the cell invasion and migration of TPC‐1 and BCPAP cells (*p* < 0.01) (Figures 6(d), 6(e)). Furthermore, Western Blot was applied to detect the expression of EMT‐related proteins. It was found that upregulated miR‐374c‐3p could suppress the N‐cadherin, vimentin, slug, and snail protein expression and promote E‐cadherin expression, thereby inhibiting EMT (Figure 6(f)).

FIGURE 6Effect of VCAN‐AS1 and miR‐374c‐3p on TC cell proliferation, invasion, migration, and EMT. (a) RT‐PCR assay was applied to validate miR‐374c‐3p overexpression in TC cells. (b), (c) CCK‐8 detecting the impact of miR‐374c‐3p overexpression on the proliferation of VCAN‐AS1‐overexpressed TC cells. (d) Cell scratch assay examining the impact of miR‐374c‐3p‐mimic on the migration of VCAN‐AS1‐overexpressed TC cells. (e) Trans‐well assay determining the impact of miR‐374c‐3p‐mimic on the invasion of VCAN‐AS1‐overexpressed TC cells. (f) Western Blot detecting the impact of miR‐374c‐3p‐mimic on the EMT‐related protein expression of VCAN‐AS1‐overexpressed TC cells. Data in the figure represent mean ± standard deviation, ^∗^ *p* < 0.05, ^∗∗^ *p* < 0.01, ^∗∗∗^ *p* < 0.001, ^##^ *p* < 0.01, ^###^ *p* < 0.001, *n* = 3.

**Figure:**
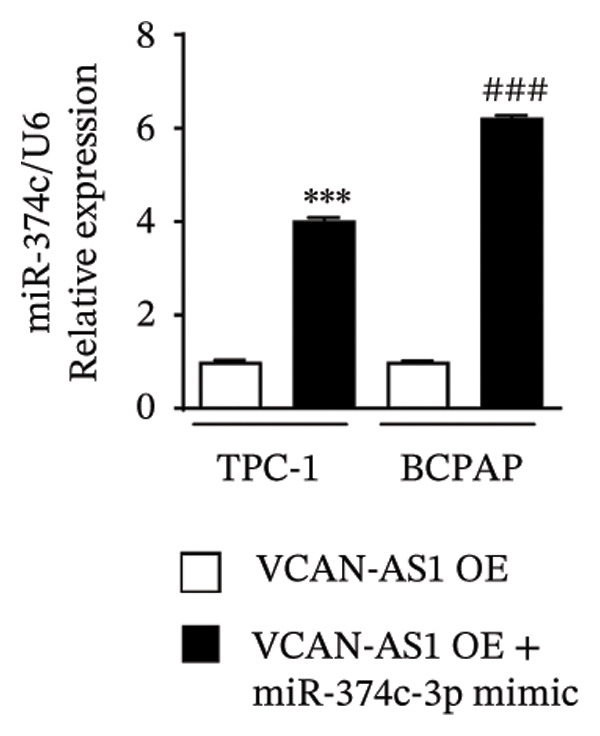
(a)

**Figure:**
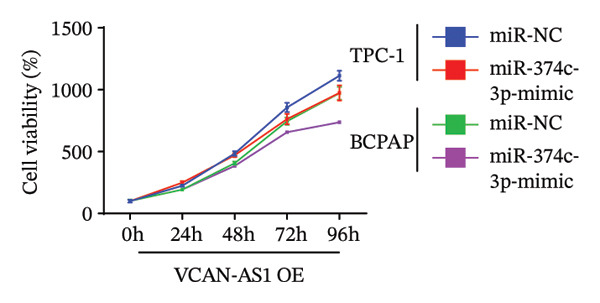
(b)

**Figure:**
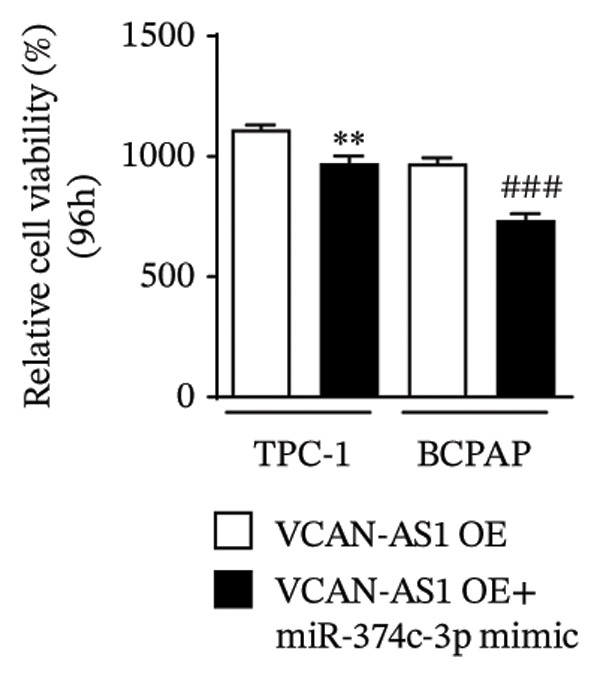
(c)

**Figure:**
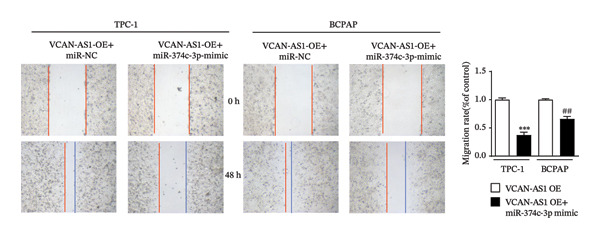
(d)

**Figure:**
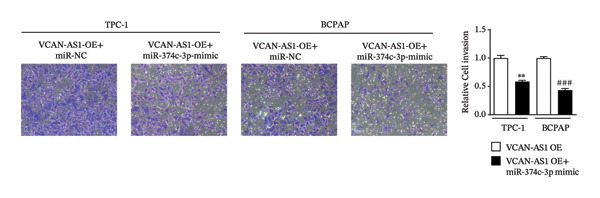
(e)

**Figure:**
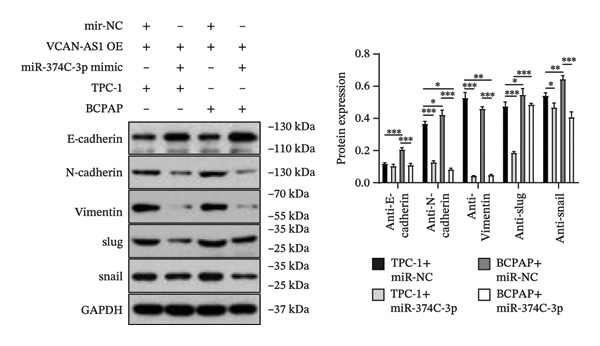
(f)

### 3.7. Effect of VCAN‐AS1 and miR‐374c‐3p on TC in Mice

To further confirm the roles of VCAN‐AS1 and miR‐374c‐3p in TC, in vivo experiments were conducted to validate the results obtained from in vitro experiments. First, Figures 7(a), 7(b) present gross images of the mice and their tumors. The results indicated that overexpression of VCAN‐AS1 significantly enhanced tumor growth, as shown by the tumor weight (Figure 7(c)) and volume (Figures 7(d), 7(e)), while overexpression of miR‐374c‐3p effectively inhibited tumor growth induced by VCAN‐AS1 overexpression. Figure 7(f) demonstrates that there were no significant differences in the body weights of the mice across the experimental groups. Next, to verify the relationship between VCAN‐AS1 and miR‐374c‐3p in vivo, RT‐qPCR results indicated that overexpression of VCAN‐AS1 significantly reduced the levels of miR‐374c‐3p in tumor tissues (Figure 7(g)). Finally, Western blot analysis was performed to assess protein expression in the tumors (Figure 7(h)). VCAN‐AS1 overexpression significantly increased the expression of N‐cadherin, vimentin, slug, and snail, while E‐cadherin expression was markedly suppressed. Importantly, in the VCAN‐AS1‐OE + miR‐374c‐3p mimic group, overexpression of miR‐374c‐3p significantly reversed the changes in these protein expressions induced by VCAN‐AS1 overexpression (Figure 7(i)). Figure 7(j) shows the design diagram of in vivo experiments.

Figure 7Effect of VCAN‐AS1 and miR‐374c‐3p on TC in vivo. (a) Gross images of mice in each group. (b) Gross images of tumors from each group. (c) Tumor weights collected after the mice were euthanized. (d) Tumor volumes collected after the mice were euthanized. (e) Changes in tumor volume over time. (f) Changes in mouse body weight over time. (g) RT‐qPCR analysis of miR‐374c‐3p levels in mouse tumors. (h) Western blot analysis of the expression levels of EMT‐related proteins in mouse tumors. (i) Quntification of the Western blot bands. (j) Diagram of in vivo experiments. Data in the figure represent mean ± standard deviation, compared with NC group, ^∗∗∗^ *p* < 0.001; compared with VCAN‐AS1‐OE group, *p* < 0.05, ^###^^*p*^ < 0.001, *n* = 3.

**Figure:**
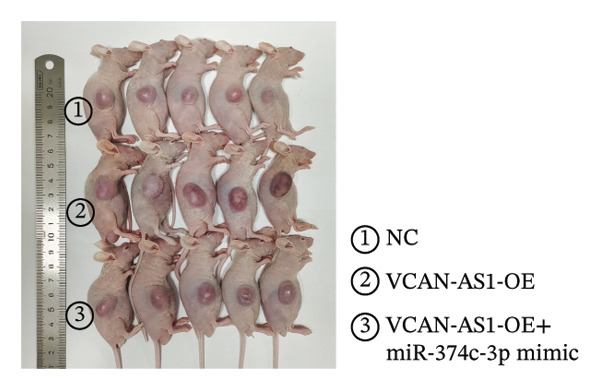
(a)

**Figure:**
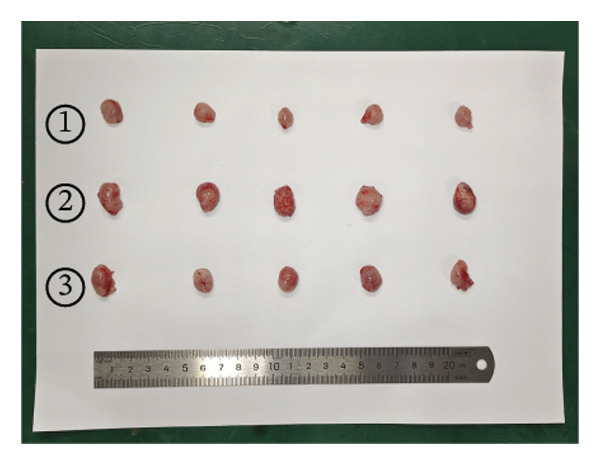
(b)

**Figure:**
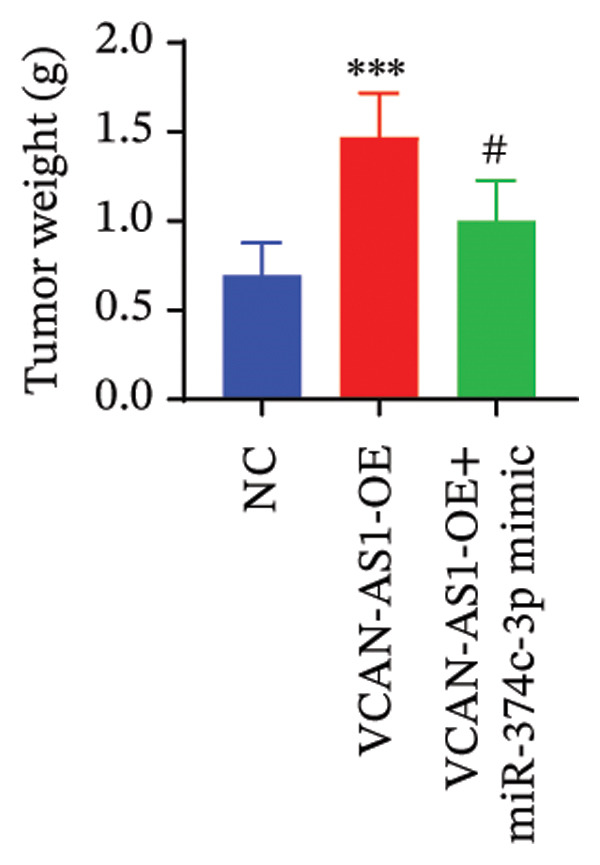
(c)

**Figure:**
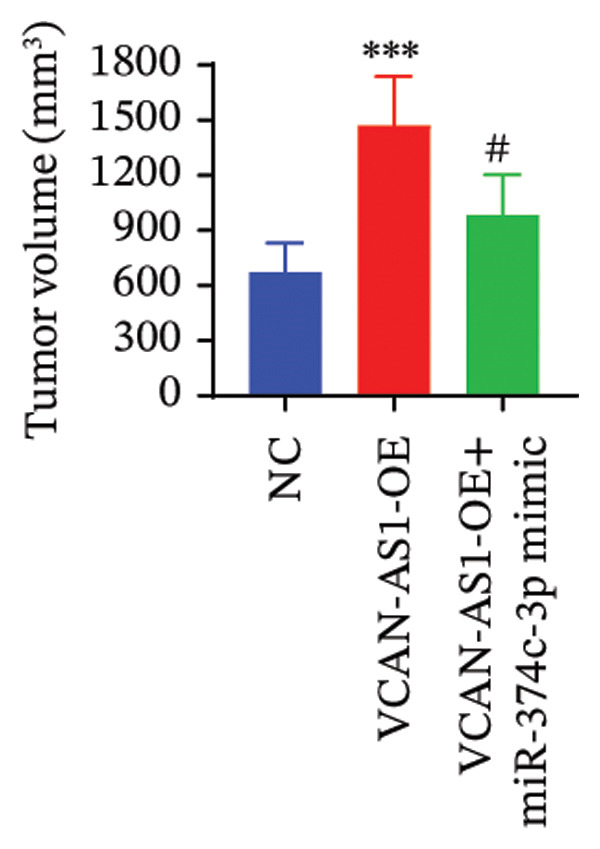
(d)

**Figure:**
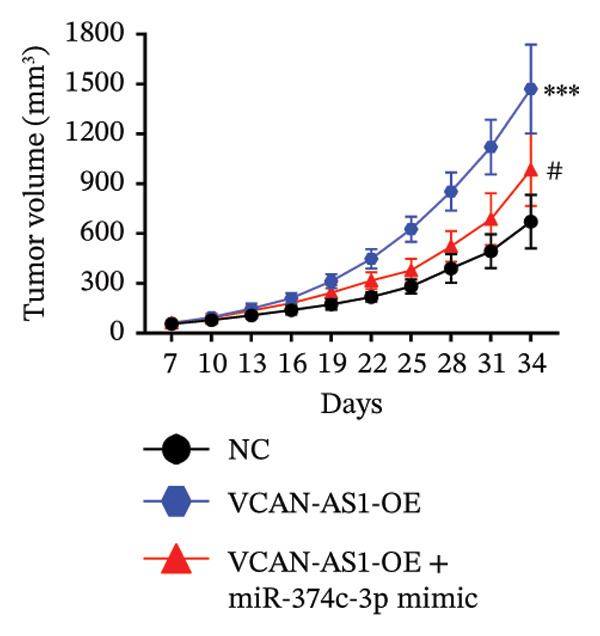
(e)

**Figure:**
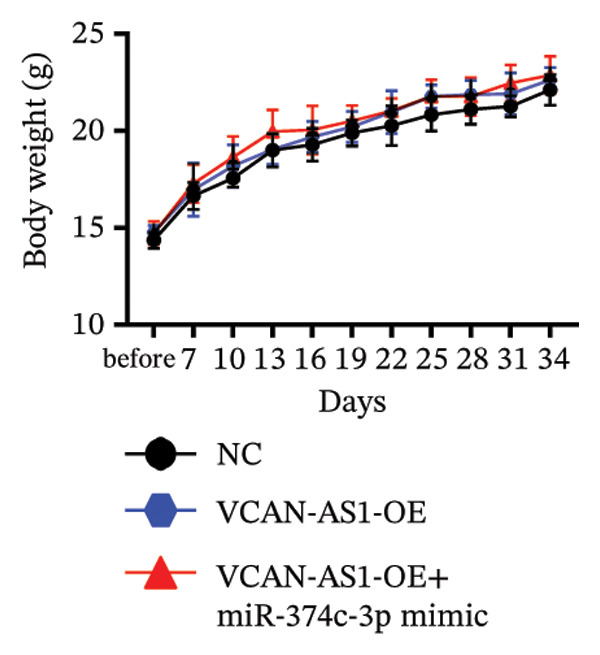
(f)

**Figure:**
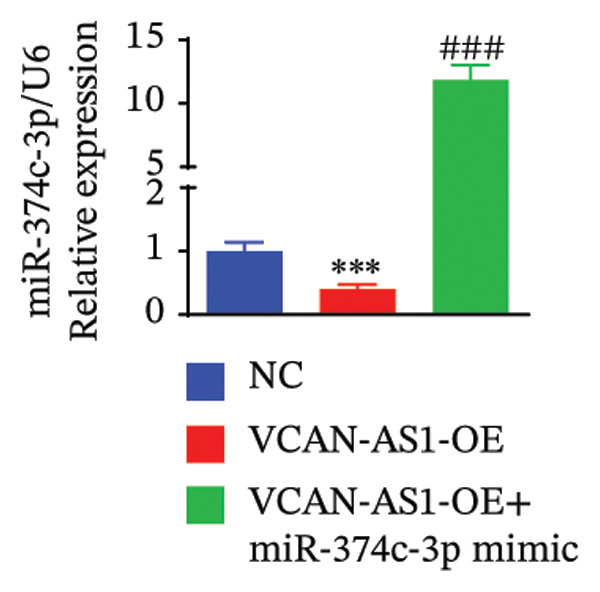
(g)

**Figure:**
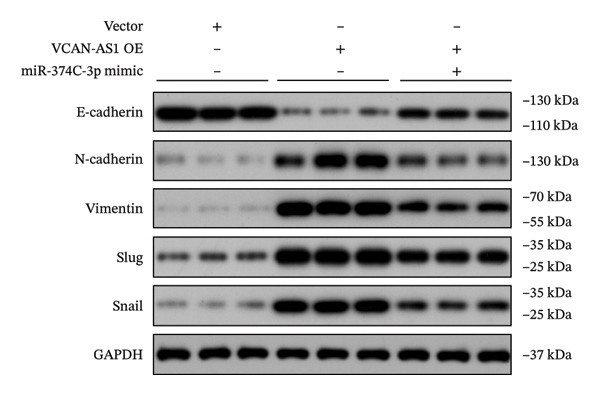
(h)

**Figure:**
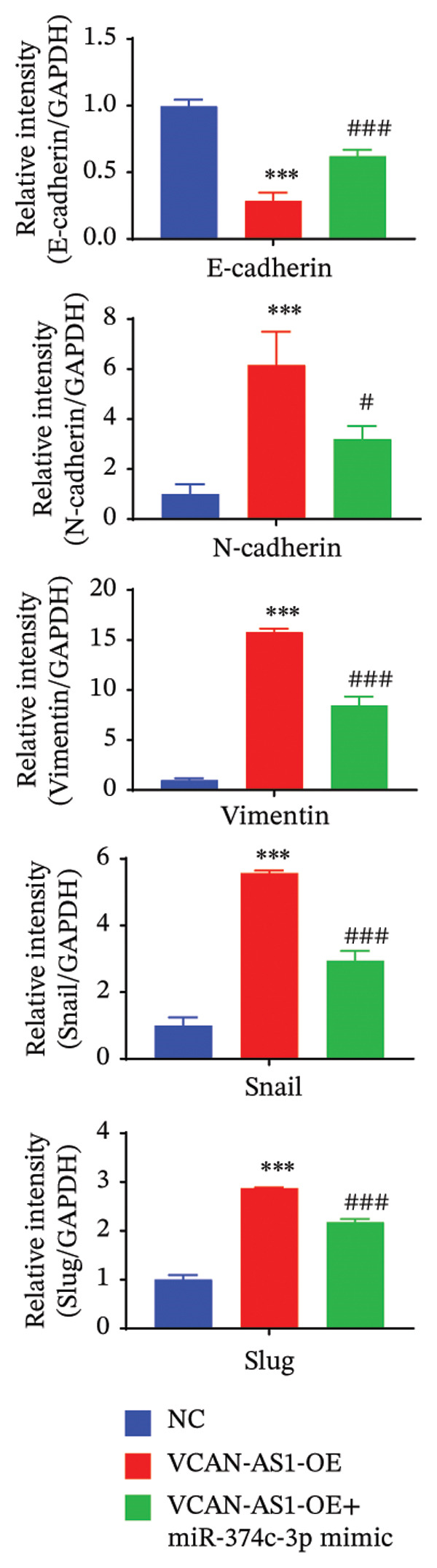
(i)

**Figure:**
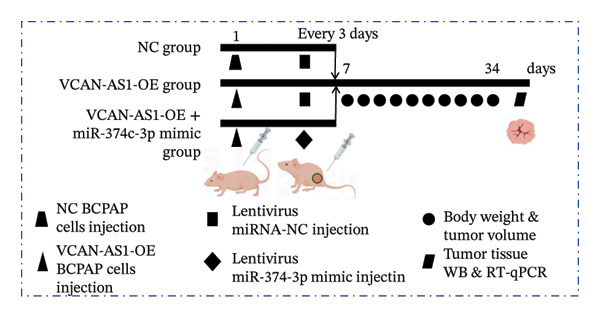
(j)

## 4. Discussion

In the present study, we performed RNA sequencing on TC tissue and adjacent normal tissue, and screened for differentially expressed lncRNAs. Following confirmation via RT‐qPCR, we focused on VCAN‐AS1 and examined its involvement in TC development, as well as explored the differentially expressed miRNAs associated with VCAN‐AS1 to better comprehend its underlying mechanisms. Our results indicated that lncRNA VCAN‐AS1 could promote TC, and silencing VCAN‐AS1 could inhibit TC cell viability, proliferation, invasion, migration, and EMT. In addition, VCAN‐AS1 acted as a ceRNA by sponging miR‐374c‐3p, thereby promoting the proliferation and metastasis of TC cells.

In recent years, the abnormal expression of lncRNAs in tumors has been the focus of much attention. Lan et al. [16] performed a genome‐wide analysis on lncRNAs in TC, while Yang et al. [17] screened for dysregulated lncRNAs in TC using microarray analysis. The aforementioned methods for screening lncRNA allow for the exploration of genes that are aberrantly expressed during the disease process. Liao et al. [9] used bioinformatics and comprehensive analysis to screen for the differentially expressed gene VCAN‐AS1 from gastric cancer tissues, and Feng et al. [7] revealed that VCAN‐AS1 could promote gastric cancer. In this study, RNA sequencing was conducted on tissue samples from patients with PTC and adjacent healthy thyroid tissue, leading to the identification of several differentially expressed lncRNAs. Notably, VCAN‐AS1 exhibited the most significant upregulation. The elevated expression of VCAN‐AS1 in TC tissues was further validated through RT‐PCR. Subsequently, miR‐374c‐3p, showing the most significant differential expression when VCAN‐AS1 was abnormally elevated in cells, was selected and subjected to GO functional enrichment analysis and KEGG signaling pathway enrichment analysis. The results showed that the gene function of miR‐374c‐3p was majorly concentrated in the endocrine system, cancer, and signal transmission, and TC is the most common endocrine system cancer, indicating that miR‐374c‐3p was associated with the role of lncRNA VCAN‐AS1 in TC, which was also confirmed in our study.

LncRNA plays a key role in various cancers [3] and becomes a biomarker for predicting tumor invasion [18]. The abnormal expression of lncRNA in TC is related to the occurrence and metastasis of TC, and lncRNA is usually involved in the proliferation, invasion, and migration of cancer cells. For example, the overexpression of XIST in TC enhances the proliferation, apoptosis, invasion, migration, and resistance to chemotherapy and radiotherapy in TC cells [19]. In addition, a previous study indicated that SLC26A4‐AS1 was significantly downregulated in TC and its overexpression significantly inhibited the metastasis of TC cells [20]. In this work, we found that VCAN‐AS1 was aberrantly upregulated in TC, promoting TC cell viability and accelerating TC cell migration, invasion, and EMT. This suggests that VCAN‐AS1 plays an oncogenic role in the development of TC. Increasing evidence suggests that EMT events play a crucial role in the progression of various tumors and the transformation of malignant cells [21], especially when cancer cells acquire the invasive and migratory capabilities of mesenchymal cells. One major molecular mechanism leading to such events involves the inhibition of cell adhesion molecules, such as E‐cadherin, and the upregulation of N‐cadherin and vimentin in the EMT pathway [22]. For instance, overexpression of the lncRNA CASC2 inhibits the EMT process in PTC cells by increasing E‐cadherin expression while downregulating the expression of ZEB1 and N‐cadherin [23]. The lncRNA TUG1 regulates the proliferation, migration, and EMT formation of TC cells by targeting miR‐145 [13]. In this study, we examined the aberrant expression of epithelial and mesenchymal markers. The results demonstrated that the overexpression of VCAN‐AS1 significantly inhibited the expression of E‐cadherin while increasing the expression of N‐cadherin and vimentin. Moreover, it elevated the expression of transcription factors slug and snail, both of which can suppress E‐cadherin transcription. This suggests that VCAN‐AS1 is involved in regulating the EMT in TC, thereby influencing the tumorigenesis and progression of TC. Notably, although VCAN‐AS1 was significantly upregulated in clinical tumor tissues, comprehensive transcriptome‐wide analyses focusing on EMT‐related gene programs in patient samples were not performed in this study. Therefore, while our in vitro and in vivo data provide functional evidence that VCAN‐AS1 promotes EMT through modulation of key epithelial and mesenchymal markers, the extent to which elevated VCAN‐AS1 expression contributes to global EMT‐associated transcriptional alterations in clinical specimens remains to be further elucidated. Future studies integrating transcriptomic profiling of patient samples will be valuable for clarifying the clinical relevance of VCAN‐AS1‐mediated EMT regulation.

MiRNAs are another type of ncRNA that are involved in regulating the expression of target genes in various cancers [24] and are considered a promising biomarker for cancer detection [25–27]. Members of the miR‐374 family play an integral regulatory role in various cancers [28] and are abnormally expressed in hepatocellular carcinoma [29], gastric cancer [30–32], and colon cancer [33], where their upregulation promotes tumor cell progression. It is widely accepted that lncRNA‐miRNA interactions play an important role in tumor development. Studies found that lncRNAs can compete with miRNAs to affect mRNA expression, thereby regulating protein expression through the lncRNA–miRNA–mRNA regulatory network. In this study, we observed the upregulation of VCAN‐AS1 through sequencing, which suppressed miR‐374c‐3p expression. In addition, RT‐qPCR validation confirmed that VCAN‐AS1 can negatively regulate the expression of miR‐374c‐3p in TC cells. Dual luciferase assay confirmed that VCAN‐AS1 could sequester miR‐374c‐3p, indicating that VCAN‐AS1 can inhibit the expression of miR‐374c‐3p by binding to it. Overexpression of VCAN‐AS1 promoted the proliferation, invasion, migration, and EMT of TC cells. However, overexpression of miR‐374c‐3p could reverse the increase in cell proliferation, invasion, migration, and EMT caused by VCAN‐AS1 overexpression. In other words, the upregulation of miR‐374c‐3p can competitively bind with VCAN‐AS1, thereby suppressing the carcinogenic effects of VCAN‐AS1 on TC by inhibiting viability and reducing invasion, migration, and EMT of TC cells. In addition, in the in vivo experiments, tumors with VCAN‐AS1 overexpression exhibited a significantly higher growth rate compared to the control group and also promoted EMT. Injection of lentivirus‐miR‐374c‐3p mimic into the tumors markedly inhibited tumor growth and effectively reversed the EMT promotion caused by VCAN‐AS1 overexpression. Therefore, we hypothesized that during TC, upregulated VCAN‐AS1 acts as a ceRNA to limit the functional availability of miR‐374c‐3p through sequence complementarity, thereby facilitating TC progression.

MiRNA regulates target genes by forming complementary pairs with the 3′‐untranslated region (3′‐UTR), 5′‐UTR, and/or coding sequence of the target gene’s mRNA, leading to the downregulation of target gene expression [28]. For example, miR‐9, induced by MYC/MYCN, directly targets the mRNA CDH1, encoding E‐cadherin, resulting in enhanced cell invasion and a context‐dependent EMT‐like conversion [12]. In nonsmall cell lung cancer cells, miR‐30a inhibits EMT by directly targeting the transcription repressor snail, which regulates CDH1 [34]. Hence, miR‐374c‐3p might modestly influence its expression levels by directly interacting with the 3′UTR of N‐cadherin, vimentin, slug, or snail.

VCAN‐AS1 and miR‐374c‐3p may serve as potential biomarkers for early diagnosis of TC. However, this study did not investigate their levels in the serum, and further clinical research is needed to demonstrate their potential as biomarkers. LncRNAs and miRNAs are often expressed in a cell‐ or tissue‐specific manner, making them specific therapeutic targets. Although there is currently no evidence on the impact of VCAN‐AS1 and miR‐374c‐3p on patient survival rates, recurrence rates, and responses to existing therapies, subsequent bioinformatics analyses and clinical validation could provide more specific and feasible strategies for targeting VCAN‐AS1 and miR‐374c‐3p.

## 5. Conclusion

This study presents novel insights into the role of VCAN‐AS1 in promoting TC progression. Specifically, our results demonstrate that VCAN‐AS1 upregulation significantly enhances TC cell viability, migration, and invasion, while also promoting the process of EMT. Conversely, silencing VCAN‐AS1 expression can hinder TC development, thereby highlighting its potential as a promising therapeutic target. Importantly, our mechanistic investigations reveal that VCAN‐AS1 exerts its oncogenic effects by targeting and inhibiting miR‐374c‐3p. Overall, these findings provide a new and compelling theoretical basis for the treatment of TC.

## Author Contributions

Y.Z., Y.W., and L.S. conceived and designed the study. Y.L. and X.C. performed the literature search and data extraction. Y.Z., Y.W., and L.S. drafted the manuscript. H.J. revised the manuscript. L.W. and C.W. reviewed and administrated the project.

## Funding

This work was supported by the Science and Technology Planning Social Development Project of Zhenjiang City (SH2022045) and Zhenjiang key research and development project (SH2020068).

## Disclosure

All authors read and approved the final manuscript.

## Ethics Statement

The collection of human tissue specimens and experiments, as well as the animal experiments, were approved by the Ethics Committee of the Affiliated People’s Hospital of Jiangsu University (K‐20190062‐Y). All patients included in this trial had preoperative discussions and signed informed consent forms. The animal experiments were conducted in compliance with the ARRIVE guidelines, the Animals (Scientific Procedures) Act 1986, and the NIH Guide for the Care and Use of Laboratory Animals.

## Conflicts of Interest

The authors declare no conflicts of interest.

## Supporting Information

Additional supporting information can be found online in the Supporting Information section.

## Supporting information


**Supporting Information** Supporting 1. Figure S1. Functional enrichment analysis of differentially expressed genes in thyroid cancer. (A) KEGG pathway enrichment analysis of differentially expressed genes. (B) Bubble plot of GO cellular component enrichment analysis of differentially expressed genes. Figure S2. High‐resolution images showing EMT‐associated morphological changes induced by VCAN‐AS1 overexpression in thyroid cancer cells.

**Supporting Information** Supporting 2. Table S1. Demographic and clinicopathological characteristics of the patients. Table S2. Differential expression data for miRNA sequencing.

## Data Availability

The raw data supporting the conclusions of this manuscript will be made available by the authors.
