# Supplementary material for: LncRNA VCAN‐AS1 Sponges miR‐374c‐3p to Promote Proliferation, Invasion, Migration, and EMT in Thyroid Cancer
Source: Int J Endocrinol. 2026 Mar 6;2026:8809262. doi: 10.1155/ije/8809262 (PMC12966362; doi:10.1155/ije/8809262)
Supplement: Supplementary file 1 — Supporting Information Supporting 1. Figure S1. Functional enrichment analysis of differentially expressed genes in thyroid cancer. (A) KEGG pathway enrichment analysis of differentially expressed genes. (B) Bubble plot of GO cellular component enrichment analysis of differentially expressed genes. Figure S2. High‐resolution images showing EMT‐associated morphological changes induced by VCAN‐AS1 overexpression in thyroid cancer cells. [file IJE-2026-8809262-s002.pptx]

## Slide 1
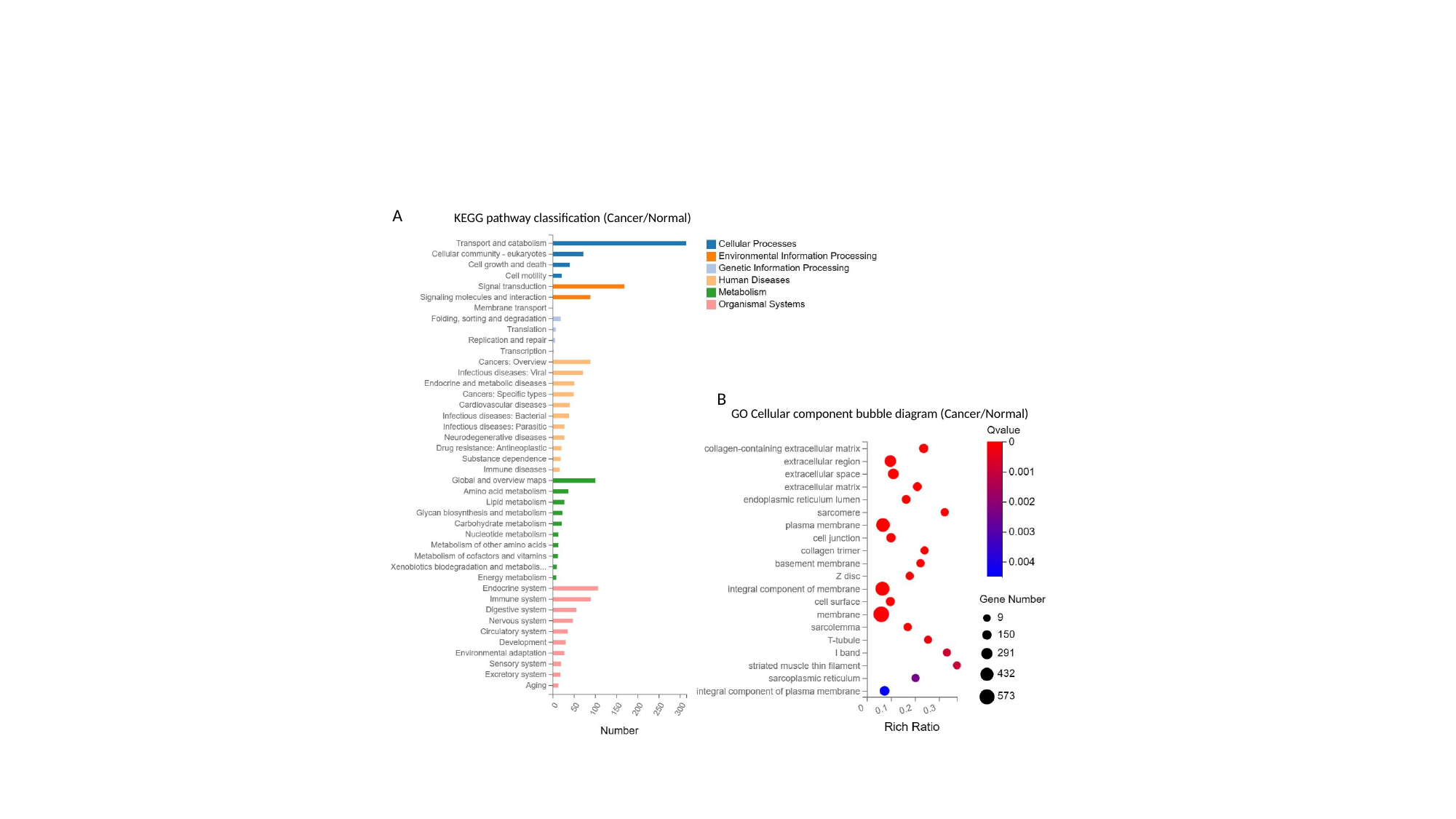

A
KEGG pathway classification (Cancer/Normal)
B
GO Cellular component bubble diagram (Cancer/Normal)

## Slide 2
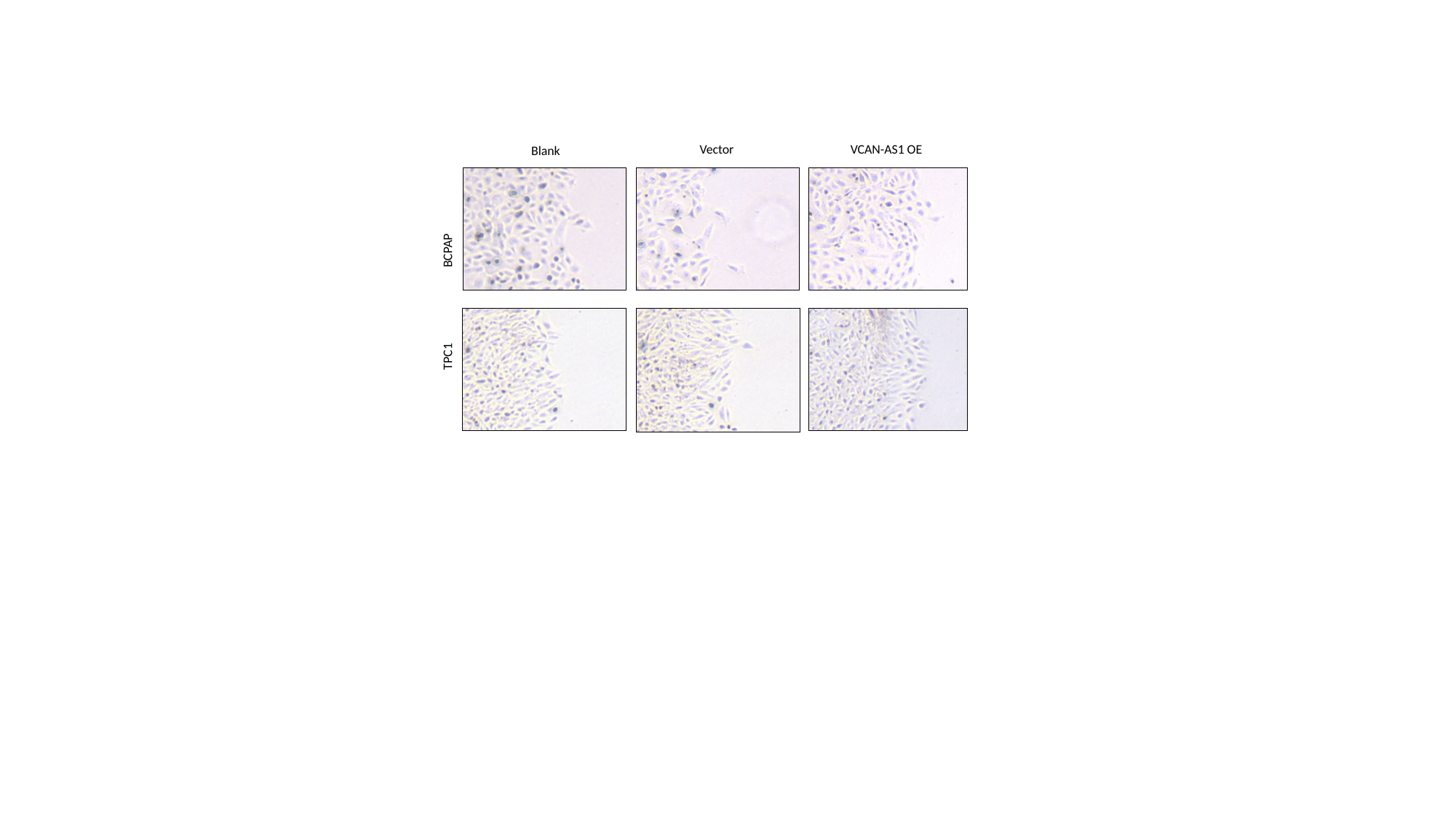

Vector
VCAN-AS1 OE
Blank
TPC1 BCPAP
