# Supplementary material for: LncRNA VCAN‐AS1 Sponges miR‐374c‐3p to Promote Proliferation, Invasion, Migration, and EMT in Thyroid Cancer
Source: Int J Endocrinol. 2026 Mar 6;2026:8809262. doi: 10.1155/ije/8809262 (PMC12966362; doi:10.1155/ije/8809262)
Supplement: Supplementary file 2 — Supporting Information Supporting 2. Table S1. Demographic and clinicopathological characteristics of the patients. Table S2. Differential expression data for miRNA sequencing. [file IJE-2026-8809262-s001.docx]

Table S1 Demographic and clinicopathological characteristics of the patients

| Patient | Age | Sex | Histology | TNM stage | Treatment before surgery |
| --- | --- | --- | --- | --- | --- |
| 1 | 30 | Female | Classic PTC | T1N1aM0 | No |
| 2 | 38 | Female | Classic PTC | T1N0M0 | No |
| 3 | 56 | Male | Classic PTC | T1N1aM0 | No |
| 4 | 46 | Male | Classic PTC | T1N1aM0 | No |
| 5 | 51 | Female | Classic PTC | T1N1aM0 | No |

Table S2 Differential expression data for miRNA sequencing

| Gene ID | Type | Ctrl_3 Expression | Ctrl_2 Expression | Ctrl_1 Expression | Treat_3 Expression | Treat_2 Expression | Treat_1 Expression | Coding Capacity |
| --- | --- | --- | --- | --- | --- | --- | --- | --- |
| hsa-miR-374c-3p | miRNA | 0.065 | 0.324 | 0.128 | 53.636 | 224.433 | 175.716 | noncoding |
| hsa-miR-4521 | miRNA | 143.65 | 150.608 | 153.16 | 204.707 | 209.209 | 218.003 | noncoding |
| hsa-miR-548ad-5p | miRNA | 0.387 | 0.259 | 0.447 | 0.768 | 4.256 | 9.385 | noncoding |
| hsa-miR-548ae-5p | miRNA | 3.548 | 11.017 | 12.577 | 0.154 | 1.037 | 0.574 | noncoding |
| novel-hsa-miR247-5p | miRNA | 49.668 | 76.795 | 52.415 | 83.092 | 151.259 | 163.828 | noncoding |
